# ForSys: Non-invasive stress inference from time-lapse microscopy

**DOI:** 10.1016/j.isci.2025.113685

**Published:** 2025-10-03

**Authors:** Augusto Borges, Jerónimo R. Miranda-Rodríguez, Alberto S. Ceccarelli, Guilherme Ventura, Jakub Sedzinski, Hernán López-Schier, Osvaldo Chara

**Affiliations:** 1 Unit Sensory Biology and Organogenesis, Helmholtz Zentrum München, Munich, Germany; 2 Graduate School of Quantitative Biosciences, Ludwig Maximilian University, Munich, Germany; 3 Instituto de Neurobiología, Universidad Nacional Autónoma de México (UNAM), Boulevard Juriquilla 3001, Juriquilla, México; 4 Systems Biology Group, Institute of Physics of Liquids and Biological Systems, University of La Plata, La Plata, Argentina; 5 School of Biosciences, University of Nottingham, Sutton Bonington Campus, LE12 Nottingham, UK; 6 The Novo Nordisk Foundation Center for Stem Cell Medicine (reNEW), University of Copenhagen, Blegdamsvej 3B, 2200 Copenhagen, Denmark; 7 Department of Biomedical Sciences, University of Copenhagen, Copenhagen, Denmark; 8 Division of Science, New York University Abu Dhabi, Abu Dhabi, Saadiyat Island, United Arab Emirates; 9 Instituto de Tecnología, Universidad Argentina de la Empresa, Buenos Aires, Argentina

**Keywords:** Optical imaging

## Abstract

During tissue development and regeneration, cells interpret and exert mechanical forces that are challenging to measure *in vivo*. Stress inference algorithms have thus emerged as powerful tools to estimate tissue stresses. Yet, effectively incorporating tissue dynamics into these algorithms remains elusive. Here, we introduce ForSys, a Python-based software that infers intercellular stresses and intracellular pressures from time-lapse microscopy. After validation, we applied ForSys to the migrating zebrafish lateral-line primordium, revealing increased stress during the cell rounding that precedes mitosis and accurately predicting the onset of epithelial rosettogenesis. We further used ForSys to study neuromast development and uncovered mechanical asymmetries linked to cell type-specific adhesion. The software performs both static and dynamic stress inference, supports command-line use, scripting, and a user-friendly graphical interface within Fiji, and accepts segmentation inputs from EPySeg and Cellpose. This versatility of ForSys enables the analysis of spatiotemporal patterns of mechanical forces during tissue morphogenesis *in vivo*.

## Highlights

• ForSys is an open-source tool to infer the mechanical states of dynamically evolving tissues
• ForSys dynamic modality outperforms static modality to recapitulate *in silico* ground truths
• ForSys predicts mechanical asymmetries linked to cell-type specific adhesion in the neuromasts
• ForSys is usable through a fiji-supported GUI, a command line interface, or through Python code

## Graphical abstract

**Figure:**
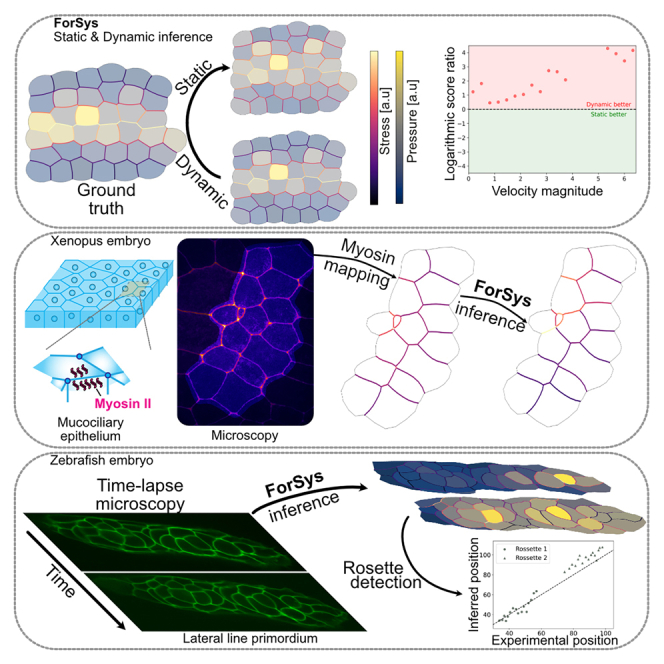

## Introduction

Recent advances in experimental techniques have reignited interest in exploring the mechanical properties of biological tissues, commonly referred to as tissue rheology. These methods have facilitated precise and quantitative measurements of tissue mechanical parameters. For example, implanted deformable magnetic droplets have been used to determine the elastic properties along the zebrafish anteroposterior axis during body elongation^1^^,^^2^ and presomitic mesoderm differentiation.^3^ Similarly, the application of optical traps has enabled the controlled deformation of cell membranes, thereby facilitating the study of viscoelastic properties during *Drosophila* development.^4^ Laser ablation experiments have also been employed in various systems to probe cortical tension by measuring the recoil of cell junctions upon laser cutting.^5^^,^^6^^,^^7^ Despite their importance, these experimental methods often come with significant drawbacks. They can be costly and necessitate specialized equipment, posing implementation challenges for many researchers. Moreover, these techniques might not be conducive to long-term imaging, potentially disrupting the normal development and, in some cases, leading to the destruction of the sample. Hence, there is a pressing need for alternative approaches to overcome these limitations while still delivering accurate and non-invasive measurements of tissue rheology.

Computational methods offer a promising alternative solution, enabling the cost-effective and straightforward implementation of tissue mechanical characterization *in vivo.*^8^ Inference techniques that use readily available microscopy images can infer the effective stress of a system based on the geometry of the cellular junctions. A key aspect of this approach centers on tricellular junctions (TJ), where three cells converge.^9^ The underlying framework relies on one major assumption: mechanical equilibrium is maintained at each TJ. The strength of these models lies in their simplicity, reducing the estimation of intercellular stresses to the solution of an overdetermined system of linear equations.^10^^,^^11^ One of the first implementations of the force-inference approach has been CellFIT,^10^ which enables the estimation of stresses from microscopy images. While CellFIT provides accurate estimates in static tissues, its applicability to dynamic tissues is limited. Also, although recent techniques using time series data offer improvements,^12^ a computational tool capable of dynamic stress inference has been lacking.

To address this gap, here we introduce ForSys, an open-source Python-based inference algorithm specifically developed to tackle the complexities of dynamical stress inference from time series experiments. By inputting microscopy images segmented with tools such as EPySeg^13^ or Cellpose,^14^ ForSys performs stress inference in either static or dynamic mode via Python scripting, a Command Line Interface, or a Fiji-based Graphical User Interface. ForSys utilizes the geometry and local velocity of cell junctions to extract the spatiotemporal stress distribution *in vivo*, providing accurate estimations of a tissue’s mechanical state.

## Results

### ForSys: A python-based open-source software to infer mechanical stress in tissues

ForSys enables the inference of intercellular mechanical stress and intracellular pressure of tissues. It takes the two-dimensional (2D) segmentation of an image, which delineates cell outlines, as its input. The input could be a skeletonized representation of the tissue (such as one generated with EPySeg^13^) or a labeled mask (for instance, generated with Cellpose^14^). ForSys then conceptualizes the entire tissue as a polygonal structure. In this structure, each polygon represents a cell, with edges connecting vertices (see “the conceptual model behind ForSys” in the STAR Methods section).

**Figure 1:**
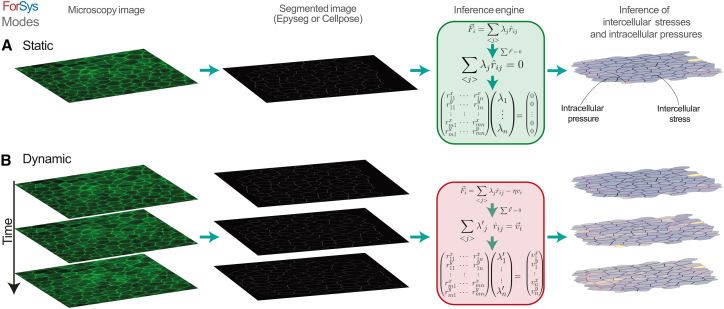
Force inference modalities of ForSys (A) The static inference is performed on a microscopy image by creating a segmented representation of the tissue. Then, ForSys reads it and builds the system of equations according to the geometrical properties of the tissue, assuming that each vertex in the tissue is in mechanical equilibrium. Lastly, the system is solved, and the intracellular pressures and intercellular stresses are inferred. (B) Similarly, the dynamical inference uses a time series of images to add dynamical information to the system of equations used in the static case, by assuming an overdamped regime. A time mesh is generated from the succession of microscopy images, and pivot vertices are tracked through time. These are the vertices at which three or more edges meet. Then, the velocity of these vertices from frame to frame is used to modify the system of equations, allowing non-static tissues to be analyzed by stress inference. Also see Figures S1–S4.

ForSys operates in two distinct modes contingent upon the input (Figure 1). When supplied with a singular segmentation of a static image, the software engages its static mode (Figure 1A) (see “statical stress inference” in the STAR Methods section). In this mode, a stress inference is applied to a single image. Conversely, if the input comprises the segmentation of a time series dataset, ForSys presents the option to function in its dynamic mode (see “the dynamic inference case” in the STAR Methods section) (Figure 1B). This mode involves the extraction of temporal trajectories for vertices from the microscopy time series, thereby incorporating corresponding vertex velocities to refine stress inference.

To improve the usability of our method, we implemented three different ways to use the software after installing the package. First, users can access the software through Python scripting by importing the ForSys package (Figure S1). Second, a Command Line Interface is available for those comfortable working in a shell environment such as Bash or PowerShell (for Linux or Windows users, respectively), eliminating the need to write Python code (Figure S2A). Finally, we developed a Graphical User Interface that integrates with Fiji (Figure S2B), providing an accessible option for users who prefer not to work with code or command-line environments. Detailed installation and usage instructions are provided in the STAR Methods section, “interacting with the ForSys software”, as well as in the software documentation.

### ForSys infers *in silico* stresses accurately in static equilibrium

To assess ForSys’s performance against existing tools, we utilized as a ground truth simulations generated by a vertex model implemented in Surface Evolver via seapipy (see “seAPIpy: generation of in silico tissues to validate ForSys” in the STAR Methods section and Figure S3), analyzed it using our software, and compared the results with outputs from previously published methods, focusing specifically on CellFIT^10^ and DLITE.^12^ Given that both tools yield similar results (Figures S5A and S5B), we opted for DLITE implementation due to its open-source nature, enabling a direct comparison with tissue stresses extracted from Surface Evolver outputs.

**Figure 2:**
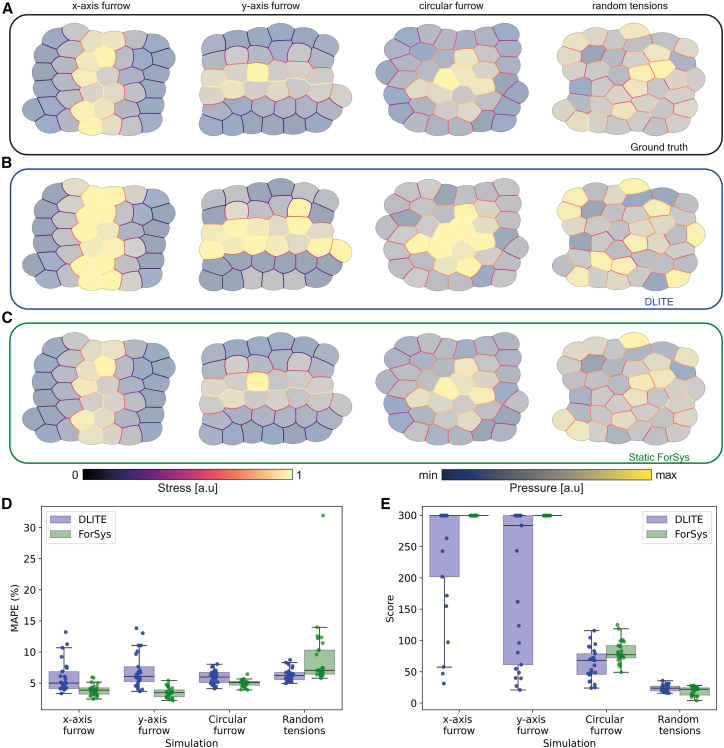
*In silico* validation of ForSys for tissues in static equilibrium Four different conditions were generated with seapipy to benchmark ForSys under the static equilibrium condition. Each column shows a representative replicate per condition at the final frame (t = 24). The ground truth (A) can be compared to the values for the DLITE predictions (B) and the static ForSys (C). The three rows shown correspond to the final frame of the simulation. The color bar above the last two panels shows the order of the colormap for both the stresses and the pressures. Pressures in the cells are represented with transparency for improved visualization. The mean absolute percentage error (MAPE) (D) and the saturated score function (E) for all simulations (*N* = 25) are represented in two boxplots, DLITE and static inference with ForSys, paired by condition. (see STAR Methods “evaluating goodness of fit”) Dots show the result for individual repetitions. In (D) and (E), each boxplot shows the median value as a horizontal bar and first to third interquartile ranges as boxes; the upper whisker is either 1.5 × the interquartile range or the maximum value (whichever is the smallest) and the lower whisker is either 1.5 × the interquartile range or the minimum value (whichever is the biggest). The pressure colormaps for the ground truth shown in panel (A), expressed as (minimum, maximum), are from left to right (0.0551, 0.3622), (0.0351, 0.3887), (0.0332, 0.0878), and (0.0289, 0.0924). For panel (B), they are (−1.17, 3.06), (−1.17, 3.01), (0.21, 1.78), and (0.38, 1.44). For panel (C) they are (−0.36, 0.71), (−0.45, 1), (−0.20, 0.38), and (−0.31, 0.31). Also see Figures S5 and S6.

We selected the final time-point (*t* = 24) of simulations generated from four different conditions to compare the ground truth from the Surface Evolver output (Figure 2A), DLITE’s estimation (Figure 2B), and ForSys in its static modality (Figure 2C). In all cases, the predicted intercellular stresses and intracellular pressures closely matched the ground truth. Moreover, both stress inference methods exhibited a high degree of accuracy and precision, as reflected by a low mean absolute percentage error (MAPE) (<10%) (Figure 2D) and a high saturated score function (∼30) (Figures 2E and S5C). Importantly, ForSys showed a significantly lower MAPE (*p* = 1e-05; *p* = 1e-8; *p* = 0.01, for the x-furrow, y-furrow, and circular furrow, respectively), higher saturated score (*p* < 0.001; *p* < 1e-4; *p* < 0.01 for the x-furrow, y-furrow, and circular furrow, respectively), and smaller interquartile range than DLITE, for all cases except the random tensions (See the “statistical estimators” section in STAR Methods for details).

These results indicate that ForSys’s static modality yields higher accuracy and precision estimations than DLITE while effectively capturing the *in silico*-generated ground truth spatial distributions in static equilibrium. Consequently, only ForSys in its static modality will be used hereafter for comparison with a static solution.

### ForSys stress inference in dynamical tissues outperforms static methods *in silico*

With the aim of inferring stress in dynamic tissue, we assumed that the tissue goes through a succession of quasistatic states in an overdamped regime,^15^ consistent with a viscoelastic response of the cell junctions to the deformations created by the forces acting on them.^16^^,^^17^ Consequently, we incorporated a viscous term proportional to the velocity of the corresponding vertex in each junction’s equation. Importantly, these velocities are not unknown: ForSys estimates them using the spatial coordinates of the vertices tracked over time. In ForSys, we call this modality of stress inference “Dynamic”.

Dynamic inference depends on a dimensionless number, which we call the Scale Parameter *ρ* (see a detailed description in the “the dynamic inference case” section of STAR Methods). Thus, we fitted *ρ* and found its optimal value for each of the examples. Our results indicate that the best dynamic results are obtained with a scale parameter of about 0.1 (see Figure S9 and STAR Methods section “determination of the scale parameter in silico”).

**Figure 3:**
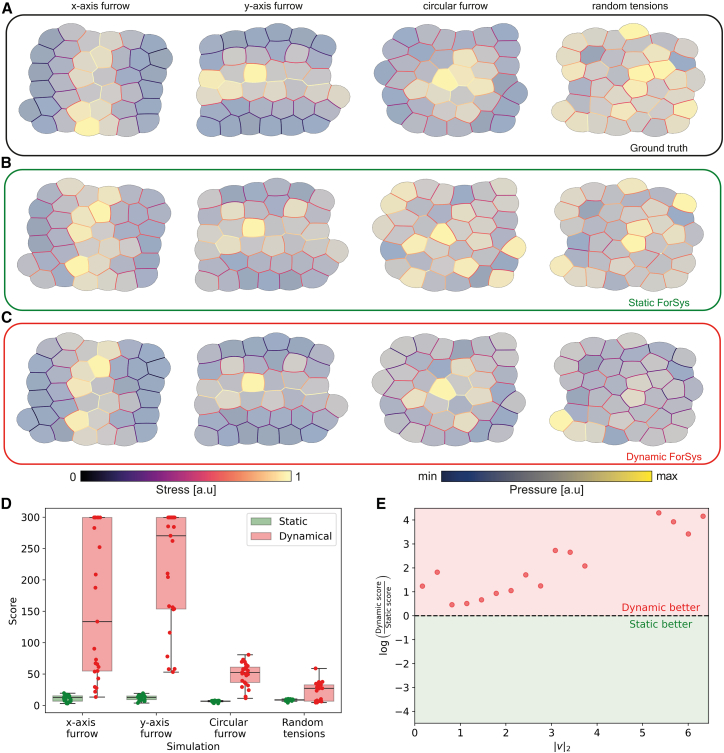
*In silico* validation of ForSys for tissues in dynamical equilibrium (A–D) We generated four examples with seapipy to test dynamical equilibrium conditions. Each column shows a representative repetition per example. The first row (A) shows the ground truth values for the stress and the pressures, the static inference made by ForSys is in the second row (B), and the dynamical ForSys inference is in (C). We show each example at one time point after the system’s tensions changed. The color bars below the last two panels show the colormap for both the stresses and pressures. The saturated score function values (D) for all simulations are represented in two boxplots, static and dynamical inference, paired by condition. Dots show the result for individual repetitions and each boxplot shows the median value as a horizontal bar and first to third interquartile ranges as boxes; the upper whisker is either 1.5 × the interquartile range or the maximum value (whichever is the smallest) and the lower whisker is either 1.5 × the interquartile range or the minimum value (whichever is the biggest). (E) Dynamic to static score function ratio ( $r=\log\left(\frac{dynamic}{static}\right)$) as a function of the $|v{|}_{2}$ bin. A ratio bigger than zero shows that the dynamic solutions performed better (Red zone), and a negative value (Green Zone) favors the static solution. The black dashed line at y = 0 separates both zones. All velocity bins favor the dynamic solution. Pressure colormaps have the following values, (minimum, maximum), for each of the panels, from left to right: For panel (A) they are (0.0609, 0.4209), (0.0488, 0.3777), (0.0285, 0.0822), and (0.0384, 0.0946); for panel (B) they are (−0.21, 0.49), (−0.33, 1), (−0.20, 0.0.20), and (−0.24, 0.25); for panel (C) they are (−0.21, 0.49), (−0.26, 0.8), (−0.19, 0.31), and (−0.23, 0.42). Also see Figures S7–S9.

Under our prescribed conditions (Figure 3A), ForSys in its static modality (Figure 3B) is outperformed by dynamic inference (Figure 3C), accurately reproducing stress and pressure distributions akin to the ground truth. Furthermore, our results indicate that dynamic modality improves static modality accuracy and precision, as indicated by lower MAPE (*p* < 1e-09; *p* < 1e-9; *p* < 1e-7, for the x-furrow, y-furrow, and circular furrow, respectively) (Figure S7A) and a higher saturated score function (*p* < 1e-08; *p* < 1e-9; *p* < 1e-9; *p* = 0.03, for the x-furrow, y-furrow, circular furrow, and random, respectively) (Figure 3D) (See the “statistical estimators” section in STAR Methods for details).

Interestingly, the accuracy and precision of stress inference in each ForSys modality are damped by the increases of TJ local movements, here reflected in the norm of the velocity vector ($|v{|}_{2}$) (Figures 3E and S7D). Notably, the dynamic modality outperforms the static one for all TJ velocities, as observed by the time evolution of MAPE (Figure S7D). This can be evidenced through the logarithmic ratio between dynamic and static scores (Figure 3E), where values greater than zero mean that the dynamic modality outperforms the static one. The outperformance of the dynamical modality is clearer for higher TJ velocities (Figures 3E, S7D, and S8). Thus, ForSys, in its dynamic modality, can retain a better approximation due to its use of the vertices' velocity, i.e., future positions, to estimate the stress.

In this section, we have shown through *in silico* validation that the dynamic modality of ForSys outperforms other methods in accurately inferring stresses in remodeling tissues.

### ForSys validation *in vivo* using the mucociliary epithelium of *Xenopus* embryos

**Figure 4:**
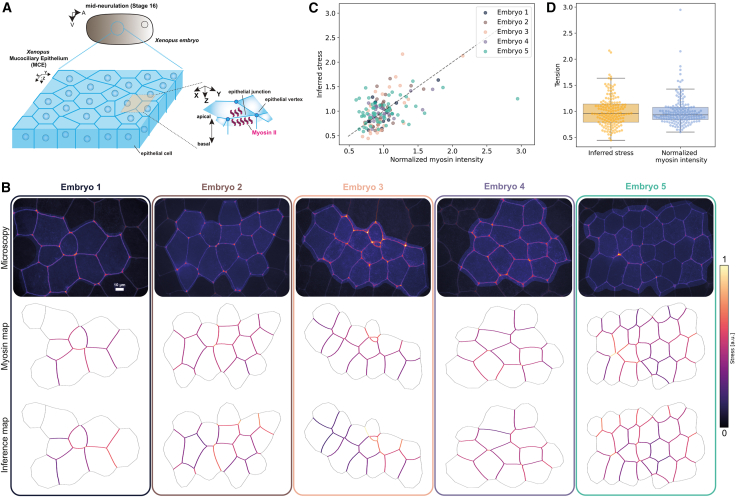
Comparison of ForSys-derived stress with myosin II measurements in the Xenopus embryo mucociliary epithelium (A) Scheme of the Xenopus embryo and position of the mucociliary epithelium. (B) Five examples of inference in Xenopus embryos. The 10 μm scale bar present in the Embryo 1 microscopy also applies to the other 4 images. The microscopy image is shown alongside the myosin intensity map and the ForSys inference result. The color code in the maps represents the myosin sensor intensity and the stress prediction. The scale was saturated at tension values of one, using the “absolute” normalization provided in the ForSys package. The highlighted region in the microscopies shows the area that was analyzed. (C) Relationship between myosin sensor intensity and stress inferred for the five examples. Each scatter point shows the value for a particular membrane in that example. The dashed black line represents the y = x line. Each color coincides with the rounded rectangle around the embryo and its font color in panel (B). The average Pearson correlation coefficient is R = 0.56 ± 0.11; (mean ± std). (D) Quantification of stresses and myosin sensor intensity for the five examples. The Inferred stresses and myosin intensities are not significantly different from each other (*p* = 0.76, Mann-Whitney U test; *p* = 0.97, Wilcoxon signed-rank test; *N* = 154). The boxplots show the median value as a horizontal bar and first to third interquartile ranges as boxes; the upper whisker is either 1.5 × the interquartile range or the maximum value (whichever is the smallest), and the lower whisker is either 1.5 × the interquartile range or the minimum value (whichever is the biggest).

To test ForSys in a biological setting, we used published data from the mucociliary epithelium in *Xenopus* embryos (Figure 4A).^18^ We quantified myosin II intensity using a non-muscle myosin II A-specific intrabody (SF9-3xGFP, for simplicity referred to as myosin II), which has been previously used as a proxy for active myosin II.^19^^,^^20^ We segmented the microscopy images using EPySeg^13^ (see STAR Methods “SF9 myosin II sensor intensity measurements” for details) and compared myosin II measurements with the stress values inferred by ForSys.

As before, we qualitatively compared the derived stress distribution maps with the ground truths, here given by the normalized myosin II sensor intensity (Figure 4B). We observed a good qualitative agreement between inferred stress and myosin intensity, with regions of higher myosin fluorescence corresponding to higher inferred stress, most noticeable in Embryo 3 and Embryo 5 of Figure 4B. In contrast, in Embryo 4 of the same panel, ForSys can reproduce a more homogenous distribution along the tissue. On a quantitative level, we found that ForSys predictions are moderately correlated with the myosin measurements for each embryo (R = 0.56 ± 0.11; mean ± std) (Figure 4C). In addition, ForSys stress predictions have a MAPE value of (21 ± 5)% (mean ± std). To further test the similarities between the myosin intensity and ForSys predictions, we pooled together all the membranes from the embryos shown in Figures 4B and 4C and compared their distributions. We found that myosin intensity and stress distributions are not significantly different (Figure 4D; *p* = 0.76, Mann-Whitney U test; *p* = 0.97, Wilcoxon signed-rank test; *N* = 154).

Altogether, ForSys in its static modality, recapitulates the stresses present in the mucociliary epithelium of the Xenopus embryo, as measured by the fluorescence of the myosin II sensor.

### Dynamic stress inference of collective cell behavior in zebrafish

We sought to explore ForSys inferences in an *in vivo* model that mixes TJs with low and high motility. To this end, we turned to two morphogenetic processes that occur during the development and homeostasis of the zebrafish lateral line, a mechanosensory organ formed by a collection of discrete organs called neuromasts.

**Figure 5:**
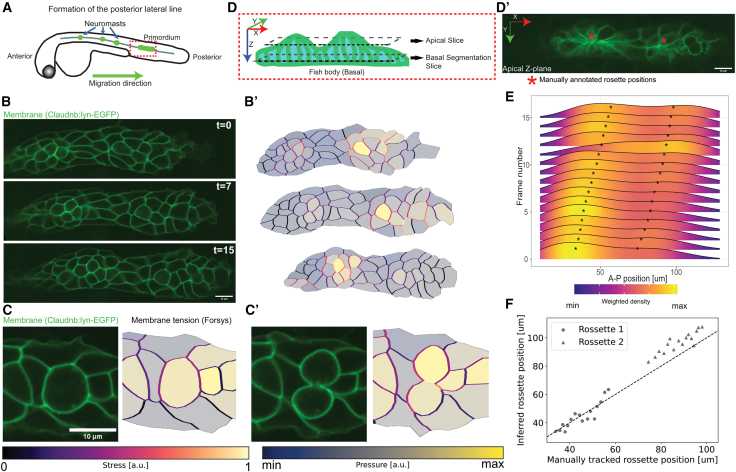
ForSys inference of a moving epithelium in the zebrafish lateral line at 2 dpf (A) Schematic of the biological model. The neuromasts of the posterior lateral line are formed by detaching from a primordium that migrates from the anterior to the posterior of the fish. (B) Frames 0, 7, and 15 of the primordium migration in which cell membranes are fluorescently marked with Claudnb:lyn-EGFP. The membrane signal is used for segmentation, which ForSys can use to predict cell membrane tension and intracellular pressure (B'). (C and C′) Consecutive planes show cell division. The membrane tension in the cell just about to divide is considerably higher than the surrounding membranes. After division, the dividing membrane retains a high tension. (D and D′) Schematic of the primordium orientation and the position of the optical planes. Constriction of the cell membranes in rosettes is evident in the apical plane. The asterisks show the anteroposterior location of rosettes. The cell segmentation was done on a Z-plane at a more basal plane (E) Ridgeline plots of Cell densities along the anteroposterior axis throughout 16 frames for a representative primordium. Time goes from bottom to top. The direction of primordium migration is to the right. The asterisks show the positions of the manually annotated rosettes. (F) Anteroposterior position of the manually tracked rosette against the inferred position by taking the local maxima of the density of pressure values from (E). The diagonal line marks y = x as a reference for comparing predicted and manually annotated values. Scale bars represent 10 μm. Tension and pressure scales are shown at the bottom of the figure. Pressure scale values (minimum value and maximum value) for panel (B′) are (−0.63, 1.6), (−0.75, 1.24), (−0.76, 1.37) for t = 0, 7, and 15, respectively. Minimum and maximum values for the pressure scale in panel (C) is (−0.74, 1.08) and (−0.85, 1.15) for panel (C′).

We first applied ForSys to an *in vivo* model of collective cell morphogenetic behavior leading to the formation of epithelial rosettes in the lateral line primordium of developing zebrafish. The primordium is a collection of just over 100 cells that move collectively from head-to-tail of the fish embryo (Figure 5A). During migration, groups of approximately 25 trailing cells periodically detach from the primordium, sequentially giving rise to individual neuromasts that are then deposited at a semi-regular pace.^21^ Although the lateral line primordium has been extensively characterized genetically,^22^^,^^23^ the evolution of mechanical forces during migration and rosettogenesis remains underexplored.

Therefore, we decided to use ForSys in its dynamic modality to analyze time lapse data of migrating primordia, whose cells’ plasma membranes were fluorescently labeled with EGFP. Migrating primordia were followed for 30 min with a temporal resolution of 2 min (Video S1) (Figures 5B and 5B′) (see a detailed description in the “zebrafish primordium migration experiments” section of STAR Methods).

Video S1. ForSys dynamic inference in the migratory primordium Top panel shows the time-lapse of the migrating primordium of Figure 5 with cell membranes marked with EGFP. The primordium migrates from anterior (left) to posterior (right). The individual frames were segmented and used as input to Forsys. Bottom panel presents the corresponding values of intercellular stresses and intracellular pressures for each frame as inferred by ForSys. The color of the cell borders and cell area represent the values of the stress and pressure, respectively.

ForSys predicted the mitotic division of primordial cells by revealing high stress in the pre-dividing cellular membrane relative to the membrane of the non-dividing surrounding cells (Figure 5C). The stresses remain partially conserved after division, mainly in the cell membrane separating the resulting cell siblings (Figure 5C’).

We then applied ForSys to predict stress tissue-wide. Apical constrictions of epithelializing cells are mechanistically associated with the formation of the rosettes that preempt neuromast morphogenesis.^24^ The apical constriction is readily detectable by morphology when looking at the apical plane of the primordium (Figures 5D and 5D′).^25^ The relationship between apical constrictions and forces in more basal planes of the cells and how they relate to rosettogenesis remains undefined.

To begin to address this possible relationship, we used time series data and aggregated the position of the cells along the anteroposterior axis of the primordium by kernel density estimation. We weighed each cell using the intracellular pressure inferred from ForSys, which results in a smoothed curve estimating intracellular pressure along the migration axis (Figure 5E). This analysis showed that the anteroposterior positions of the rosettes, manually annotated by looking at apical constriction (Asterisks in Figures 5D’ and 5E), correlate with the predicted zones of high intracellular pressure in the basal plane inferred by ForSys. The closeness between the predicted pressure maxima and the manually annotated rosette formation indicates a high correlation between these two quantities during primordium migration (R = 0.99, *p* < 1e-51, *N* = 61; for rosettes 1 and 2 combined) (Figure 5F).

**Figure 6:**
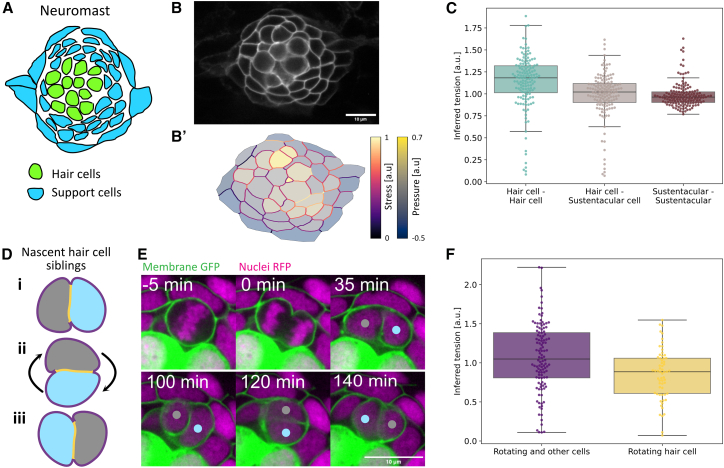
*In vivo* ForSys inference in an epithelium with rotating cells (A) Schematic of cell composition in a zebrafish lateral line neuromast. Sensory hair cells are located in the center and are surrounded by support cells. (B) Image of a neuromast whose cells can be tracked by membrane-tethered EGFP. (B′) ForSys tension and pressure inference after membrane segmentation. (C) The tension inferred for membranes is classified by the type of cell-cell contact. The homotypic contacts between hair cells show the highest predicted tension, while the homotypic contacts between support cells show the lowest on average. Each data point is the mean of the predicted tension values for each membrane type in one frame. The frames come from *N* = 7 time-lapse experiments. (D) Schematic of the planar cell inversions occurring in 50% of the nascent hair cell pairs: sibling hair cells perform a 180° rotation to exchange positions along the anterior-posterior axis. The coloring of membranes corresponds to the colors of the respective boxplot in panel (F). (E) Time-lapse frames show the *in vivo* rotation process: around 100 min after mitosis, the nascent hair cells exchange anteroposterior positions by rotating in the epithelial plane. The sibling cells remain attached during the rotation, while the surrounding cells do not actively participate in the movement. The TgBAC(cxcr4b:H2B-RFP) transgenic was used to label nuclei. (F) Homotypic tensions between the young rotating hair cells are significantly lower than their contacts with the surrounding cells. In (C) and (F), boxplots show the median value as a horizontal bar and first to third interquartile ranges as boxes; the upper whisker is either 1.5 × the interquartile range or the maximum value (whichever is the smallest) and the lower whisker is either 1.5 × the interquartile range or the minimum value (whichever is the biggest). Scale bars represent 10 um.

Encouraged by our previous results, we next analyzed mature neuromasts. The center of this organ is occupied by mechanosensory hair cells, which are surrounded by non-sensory supporting cells (Figure 6A).^26^ We used a plasma membrane marker to define cells, which were segmented using ilastik^27^ and EPySeg^13^ (Figure 6B) (see a detailed description in the “zebrafish neuromasts videomicroscopy and analysis” section of STAR Methods). Then, we used the dynamical modality of ForSys to estimate the stress at each membrane (Figure 6B’) and found that membranes belonging to hair cells have higher stress on average. Homotypic interfaces between hair cells have the highest stress (*p* < 1e-7 vs. hair cell-supporting cell interfaces). On the other hand, homotypic contacts between supporting cells have the lowest stress (*p* < 0.006 vs. hair cell-supporting cell interfaces) (Figure 6C).

We then focused on a still-puzzling process called planar cell inversion (PCI).^28^^,^^29^ PCI occurs when supporting cells give rise to hair-cell progenitors, which divide once to generate a pair of hair cells. Approximately half of the resulting nascent hair-cell pairs undergo a 180° rotation around their geometric center^28^^,^^29^ (Figures 6D and 6E). The mechanical forces occurring during cell-pair inversions are not known, but the physical modeling of the cell doublet suggests that differential surface tension plays a central role.^30^^,^^31^ Therefore, we focused our analysis on the homotypic junctions between the sibling hair cells and compared them to those with the surrounding supporting or hair cells. We found that the stress in the membranes juxtaposing the rotating hair-cell pair is significantly smaller than that between hair cells and the surrounding cells (*p* < 0.0005) (Figure 6F). Because tension and adhesion are generally inversely related, PCI could be characterized by a strong adhesion within the rotating cell pair and weaker adhesion with the surrounding cells. This result suggests a cell-type and cell-state-specific adhesion pattern that influences contact remodeling necessary for coordinated cell-pair rotations.

Taken together, these results show that ForSys’s dynamical implementation predicts high stresses before cell division in a migrating tissue. They also revealed that rosette formation could be prefigured by mechanical rosettogenesis changes in the cells, which allows the inference of apical constrictions during rosettogenesis using information from basal planes.

### Scale parameter selection *in vivo*

**Figure 7:**
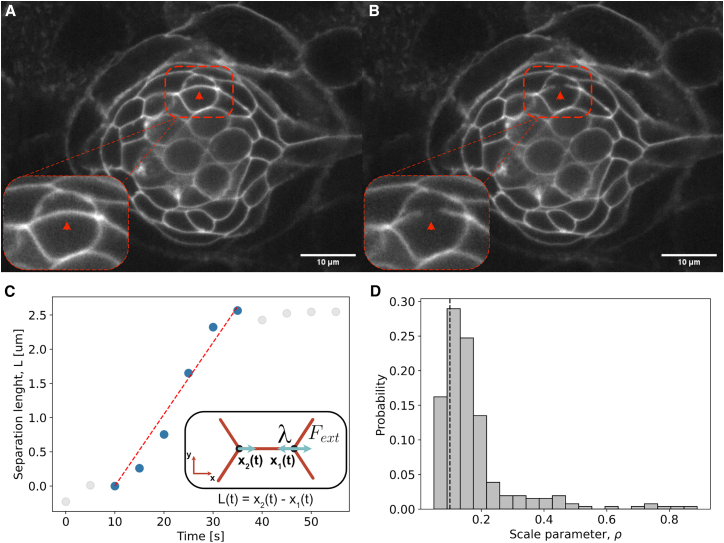
Scale parameter determination through laser ablation The scale parameter was determined through laser ablation experiments, as described in the STAR Methods section “zebrafish laser ablation experiments.” Microscopy images are taken every second, including just before (A) and just after (B) ablation. The red arrow indicates the membrane where the ablation takes place, and the dashed red line is a guide to the eye. The inset in the lower right corner of both (A) and (B) panels zooms in on the ablation zone. Scale bars represent 10 μm. (C) Example trajectory of the membrane after ablation. The y axis shows the elongation relative to the length in the frame just before ablation occurs. Blue scatter dots are used for the fitting, gray points are not. The inset in the lower right corner shows a sketch of the ablated membrane. (D) Using the linear fits from thirty-seven ablation experiments, we determine the scale parameter by multiplying each experimental slope with the characteristic velocity (v) of each of the seven neuromasts that comprise Figure 6. In panel (D), the scale parameter resulting from the fits is represented as a histogram. The vertical dashed line represents the value used in our inferences (ρ = 0.1).

The results presented in the previous section were obtained by setting the scale parameter to 0.1, the optimal value identified through *in silico* validation. To assess the validity of this default value experimentally, we performed *in vivo* laser ablation of cell membranes in zebrafish neuromasts and measured the time evolution of the distance between the pivot vertices delimiting the ablated membranes (Figures 7A–7C).

We modeled the system using a minimal physical description. For simplicity, we aligned the two pivot vertices along the x axis, with their positions defined as *x*_1_(*t*) and *x*_2_(*t*) (Figure 7C, inset). Prior to ablation, the membrane is assumed to be in mechanical equilibrium, consistent with the central assumption of ForSys. Thus, the magnitude of the net external force at each pivot vertex is balanced by the membrane stress that ould be inferred by ForSys ($\|F_{ext}\|=\lambda$; see Equation 2 in STAR Methods section “statical stress inference”). Immediately after ablation, each vertex recoils with velocity *v*, and a viscous damping force opposes this motion, balancing the membrane stress (*λ* = *ηv*; see Equation 6 in the STAR Methods section “statical stress inference”). As a result, the distance between the two vertices evolves over time as:

(Equation 1) $$L\left(t\right)=L_{0}+2\left(\lambda /\eta \right)t$$

where *L(t)* = x_2_(t) – x_1_(t) is the separation length between the vertices at time *t*, and *L*_*0*_ is their initial separation length.

By fitting this expression to the recoil dynamics measured in zebrafish neuromasts at short times, we extracted the ratio λ/*η* (Figure 7C). Using the characteristic velocity of neuromast vertices ($\bar{v}$, as defined in Equation 7 in the STAR Methods section “statical stress inference” and determined from the inferences shown in Figure 6) and applying Equation 8 in the STAR Methods section “statical stress inference”, we estimated the corresponding value of the scale parameter. The experimentally determined value of the scale parameter was 0.17 ± 0.13 (mean±std), which is consistent with the value used in our inferences (Figure 7D).

## Discussion

Here, we introduce ForSys, a software that statically and dynamically infers stresses without disrupting biological tissues. Traditional inference methods rely on geometrical information to calculate the relationship among the stresses acting on cell membranes in a static image. However, these methods generally lack the dynamical component present in time-series microscopy. ForSys extends the applicability of inference techniques by enabling dynamic stress inference in cell membranes when tissues are in motion.

We validated our software in its static and dynamic modalities with different *in silico* spatial patterns of tissue stresses using a cell-based computational model implemented in Surface Evolver,^32^ which we integrated into a Python package called seapipy.^33^ Our results show that ForSys can recover the ground truth in its static and dynamic modalities. Significantly, the dynamic modality improves the accuracy of the static modality in the tested conditions. Unlike static inference, which involves a single intrinsic scale embedded in the inferred stresses, dynamic inference introduces a viscous term proportional to the velocities of the nodes, thereby introducing a second physical scale. The interplay between these two scales is captured by the scale parameter, which modulates the relative contributions of viscous forces and other mechanical stresses in the inference process. In this sense, the scale parameter plays a role analogous to the Weissenberg number or the Capillary number.^34^^,^^35^^,^^36^ The optimal value found for this number indicates that elastic forces are an order of magnitude larger than viscous forces. Strikingly, dynamic inference outperforms static inference *in silico* even when elastic forces dominate over the viscous forces, pointing to a wide applicability of the dynamic modality.

We then tested ForSys in the Xenopus embryonic mucociliary epithelium. We found a positive correlation between the inferred stress and cortical stress that was indirectly measured using variations in the intensity of myosin II. As the development of the embryonic mucociliary epithelium progresses over several hours, continuous, direct probing of the mechanical forces is extremely laborious, interferes with tissue development, and is hardly compatible with single-cell resolution measurements. Therefore, using ForSys for the non-invasive mapping of mechanical forces at the scale of an entire tissue across time could pave the way for a more comprehensive understanding of the mechanical forces that drive tissue development.

We further demonstrated the power of ForSys by studying two aspects of organ development and homeostasis using the neuromasts of the lateral line in zebrafish embryos. Specifically, we addressed two processes that involve a complex collective cell behavior. First, we applied dynamical ForSys to the migrating lateral-line primordium. Although this process has been extensively dissected genetically, it is still unknown what forces play a role during migration and neuromast deposition.^37^^,^^38^ Therefore, this process of collective cell migration will benefit from an accessible and non-invasive method to estimate forces in a dynamical tissue. Two characteristics of this migratory primordium make it well-suited for applying ForSys: the tissue as a whole is migrating through the lateral line, and its membranes have a curved shape. We showed that ForSys can detect cell division and rosette formation. ForSys will be useful for testing various hypotheses about tissue mechanics in other dynamic cell systems, for instance, during tissue repair and organ regeneration.

We also applied ForSys to address the still mysterious process of planar cell inversion, during which sibling cells rotate around their centroid after the mitotic division of their progenitor.^28^ We discovered that homotypic contacts between rotating cells have the most stress, whereas the contacts between the rotating pair have lower stress than the contacts of each hair cell with its neighbors. This strongly suggests that adhesion dynamics during rotation are associated with strong homotypic interactions between the sibling cells and weak heterotypic interactions with the surrounding cells, enabling contact exchange during the inversion.^28^

The development and improvement of stress inference tools presents a promising avenue to capture the spatiotemporal complexity of mechanical forces during tissue morphogenesis, which circumvents challenges related to sample fixation and limited temporal resolution.^39^ An interesting possibility is to use fluorescence-based tension and strain mapping techniques, such as those employing molecular tension sensors^40^^,^^41^ or fluorescence intensity correlates^42^^,^^43^ that enable the direct visualization of mechanical forces in tissues in combination with ForSys. Future studies combining these complementary methods could provide a more comprehensive understanding of tissue mechanics *in vivo*.

ForSys provides a versatile and noninvasive tool for studying spatiotemporal patterns of mechanical stresses during tissue morphogenesis *in vivo*. This software makes stress predictions that can guide researchers in conducting further experiments, which can significantly contribute to understanding the mechanisms involved in development and regeneration. ForSys was built as open-source software in Python, thus allowing the community to participate in its development and maintenance. With its usability in mind, aside from the possibility of writing Python scripts to interact with ForSys, it has been further equipped with a Command Line Interface and a Graphical User Interface embedded in Fiji^44^ to facilitate its adoption. In our eyes, an interesting future perspective will be to extend the software to tissues in non-equilibrium conditions and adapt the method to operate within a 3D geometry to generate 4D mechanical stress inference.

### Limitations of the study

The applicability of the method described in this article, as well as the ForSys software, relies on two groups of assumptions: methodological and theoretical.

The methodological assumptions primarily concern limitations related to the quality of the input data supplied to the program. These include, but are not limited to, poor segmentation leading to the inaccurate reconstruction of cellular membranes, or the inability to track all agents contributing to the system’s movement, such as interactions with structures located outside the region of interest or out of the imaging plane. In general, the higher the quality of the input data, the more accurate the predictions produced by ForSys.

The theoretical assumptions involve the validity of the underlying hypotheses in the tissue under study. For instance, if the acceleration of tricellular junctions is sufficiently large, mechanical equilibrium may not hold, and the tissue would no longer behave as an overdamped system. However, in most biological tissues, inertial effects containing acceleration terms are negligible compared to viscous damping, supporting the overdamped regime assumption.^15^ Another theoretical limitation of our model is the assumption that membrane stresses remain constant within the time window of each image frame. If stresses fluctuate on timescales faster than the image acquisition frequency, ForSys will not capture these rapid changes. In such cases, the inferred stresses should be interpreted as averaged or representative values over the time interval.

## Resource availability

### Lead contact

Further information and requests for resources should be directed to Osvaldo Chara (osvaldo.chara@nottingham.ac.uk).

### Materials availability

This study did not generate new materials.

### Data and code availability

The seapipy^33^ codebase is available on GitHub at https://github.com/borgesaugusto/seapipy. ForSys^45^ is available on GitHub https://github.com/borgesaugusto/forsys. All data reported in this article will be shared by the lead contact upon request.

## Acknowledgments

The authors thank Luis Morelli, Fabian Rost, Nicolas Aldecoa, Alice Descoeudres, and all the members of the Chara laboratory for valuable comments and suggestions. A.B. was funded by the 10.13039/501100002347BMBF 01GQ1904 grant. A.S.C. was funded by a Doctoral fellowship from 10.13039/501100002923CONICET, Argentina, and by a 10.13039/501100000268Biotechnology and Biological Sciences Research Council (BBSRC) grant [grant number BB/X014908/1]. J.S. acknowledges the support of the 10.13039/501100009708Novo Nordisk Foundation (grant number NNF22OC0076414, NNF19OC0056962) and 10.13039/501100012331LEO Foundation (grant number LF-OC-19-000219), the 10.13039/501100000781European Research Council Consolidator Grant (ERC CoG 101125803 MechanoFate). The Novo Nordisk Foundation Center for Stem Cell Medicine (reNEW) is supported by a 10.13039/501100009708Novo Nordisk Foundation grant number NNF21CC0073729. O.C. was funded by 10.13039/501100002923CONICET, 10.13039/501100006668Fondo para la Investigación Científica y Tecnológica (PICT-2019-03828) and the 10.13039/501100000268BBSRC grant BB/X014908/1 to O.C.

## Author contributions

A.B. developed the ForSys code, simulated the in silico results, analyzed all the inference results, and wrote the article. A.S.C. contributed to the development of the ForSys code and analyzed all the inference results. G.V. generated the Xenopus images and edited the article. J.M.-R. acquired the zebrafish microscopy images and analyzed the corresponding results. J.S. supervised the Xenopus project and edited the article. H.L.S. supervised the Zebrafish project and edited the article. O.C. conceived the project, contributed to the ForSys code, analyzed all the inference results, wrote the article, and supervised A.B. and A.S.C.

## Declaration of interests

The authors declare no competing interests.

## STAR★Methods

### Key resources table

**Table:** 

REAGENT or RESOURCE	SOURCE	IDENTIFIER
**Experimental models: Organisms/strains**		

Zebrafish Tg[myo6b:actb1-EGFP]	Kindt lab	https://doi.org/10.1016/j.devcel.2012.05.022
Zebrafish Tg[-8.0cldnb:Lyn-EGFP]	Gilmour lab	https://doi.org/10.1016/j.devcel.2006.02.019
Zebrafish TgBAC(cxcr4b:H2B-RFP)	Lecaudey Lab	https://doi.org/10.1038/s41467-018-06094-4
Xenopus laevis	Sedzinski Lab	N/A

**Recombinant DNA**		

SF9-3xGFP myosin II sensor	Sedzinski lab	https://doi.org/10.1038/s41467-022-34165-0

**Software and algorithms**		

seapipy v0.2.1	Repository	https://doi.org/10.5281/zenodo.10853567
ForSys v1.1.2	This paper	https://doi.org/10.5281/zenodo.15789474
Epyseg v0.1.52	Prud'Homme Lab	https://doi.org/10.1242/dev.194589
Tissue Analyzer	Eaton lab	https://doi.org/10.1007/978-1-4939-6371-3_13
Ilastik v1.3.3	Kreshuk lab	https://doi.org/10.1038/s41592-019-0582-9

### Experimental model and study participant details

#### SF9 myosin II sensor intensity measurements

Images of stage 16 to stage 20 Xenopus embryos expressing the SF9-3xGFP myosin II sensor were acquired in using a 3i spinning disk microscope with a Plan-Apochromat ×63 oil objective (N.A. = 1.4) mounted on an inverted Zeiss Axio Observer Z1 microscope (Marianas Imaging Workstation [3i—Intelligent Imaging Innovations]), equipped with a CSU-X1 spinning disk confocal head (Yokogawa) and an iXon Ultra 888 EM-CCD camera (Andor Technology). From these images we obtained maximum intensity Z projections, which were then used to extract myosin intensity values of the epithelial junctions. After automatic segmentation with EPySeg, cell membranes were manually corrected, and the final shape was obtained by using a third-order Savitzky–Golay filter with a window of 5 pixels. For all vertices constituting a membrane in the segmentation, smoothed intensity values were first obtained by taking the median over first neighbors. The intensity value for each membrane is then defined as the mean of smoothed intensities at each of its constituting vertices. Then, to allow comparison with the inferred stresses, these values were normalized to a mean value of one for each embryo.

#### Zebrafish primordium migration experiments

Zebrafish carrying the Tg[-8.0cldnb:Lyn-EGFP]^46^ were kept under standard conditions at 28.5°C. All zebrafish experiments were performed in accordance with protocols approved by the Ethical Committee of Animal Experimentation of the Helmholtz Zentrum München, the German Animal Welfare act Tierschutzgesetz §11, Abs. 1, Nr. 1, Haltungserlaubnis according to the European Union animal welfare, and under protocol number Gz.:55.2-1-54-2532-202-2014 and Gz.:55.2-2532.Vet_02-17-187 from the “Regierung von Oberbayern” (Germany).

At 40-48 hours post-fertilization, larvae were anesthetized with 1.5% MS222 in Danieau Buffer and mounted in 0.8% low-melting point agarose on a glass-bottom petri dish. Larvae were imaged in a custom-built Zeiss inverted spinning disk confocal microscope. 16 slices Z stacks of the migrating primordium II were acquired every two minutes with a 63X objective. Subsequently, one z-slice was manually selected from each frame, and the membrane image was segmented using Tissue Analyzer.^47^ The image segmentations were used for ForSys predictions, and the cells' centroids' X and Y coordinates, the time point (frame number), and the cell pressures were exported. The probability density function of the cell position along the anteroposterior axis was estimated via a Gaussian kernel in the R statistical software. The value of cell pressure was used as a weight in the density estimation. From this density curve, local maxima were determined by an *ad-hoc* algorithm implemented in *R*, which compares the sign of the first and second derivatives.

#### Zebrafish neuromasts videomicroscopy and analysis

The videomicroscopy data previously published by Kozak and colleagues was acquired as described and re-analyzed.^28^ Zebrafish larvae (*Danio rerio*) carrying the transgenics myo6b:β-actin-GFP^48^ and *Tg[−8.0cldnb:Lyn-EGFP]*^46^ were anesthetized in MS222 and mounted in 1% low-melting point agarose. Larvae were imaged simultaneously using a Zeiss custom-built inverted spinning-disc confocal microscope with a 63× water-immersion objective. Stacks of 16-20 *z*-slices 1 μm apart were acquired every 200 s. Hair cells can be identified by the myo6b:β-actin-GFP staining in the apical side, while *Tg[−8.0cldnb:Lyn-EGFP]* marks all neuromast membranes and allows cell segmentation. In total, 14 hair cell divisions were included in the analysis, and partitioned into the “inverting” or “non-inverting” group by whether the final position of the sibling hair cells is the opposite with respect to the anteroposterior axis.^28^ All 4D movies were processed using FIJI software.^44^ Stacks were centered by laying point regions of interest at time frames of significant drift and then running the Manual Drift Correction plugin and then further registering for *z-*slice drifts using the plugin Correct 3D drift.^49^

After registration, one *z*-slice per time point was selected to segment membranes, using the Autocontext workflow from ilastik v1.3.3.^50^ The resulting probabilities were loaded in the multicut segmentation workflow to get a skeletonized segmentation of cells. These automated segmentations were loaded into TissueAnalyzer^47^ and manually corrected and semi automatically tracked. From the software Tissue analyzer, we exported two types of data: (1) cell tracking data containing *x* and *y* centroid position, cell area, perimeter (in pixels) and an ID identifying individual cells through time; (2) bond tracking data, indicating the identity of cells sharing a membrane segment, and the length of the membrane segment (in pixels).

The same segmentation images were then loaded into ForSys for stress inference and merged with the Tissue Analyzer data thereby getting an integrated dataset where it is known the identity of each cell, its pressure inference, and the membrane tension estimation for each membrane segment.

For Figure 6E, fish additionally carrying the transgenic TgBAC(cxcr4b:H2B-RFP) were used to label nuclei.

#### Zebrafish laser ablation experiments

Zebrafish larvae Tg[−8.0cldnb:Lyn-EGFP] 5 days post fertilization were prepared identically and imaged in the same microscope as described in STAR Methods section “zebrafish neuromasts videomicroscopy and analysis”. The laser ablation was performed with a iLasPulse laser system (Roper Scientific SAS, Evry, France) mounted on a Zeiss Axio Observer inverted microscope equipped with a 63× water objective lens., as described in previous works.^51^^,^^52^ Thirty-seven neuromasts were ablated with only one ablation per fish. For each ablation, the microscope was centered in a L2 or L3 neuromast. Time Lapse movies were taken with a frequency of one image per second. Three laser pulses (355 nm, 400ps) were automatically triggered between the 3rd and 4th frame to acquire microscopy images before and after the ablation of the membrane in a small circular ROI in the mid part of the cellular membrane (Figures 7A and 7B). We manually tracked the position of the tricellular junctions belonging to the ablated membrane as a function of time. We used the membrane’s recoil as a function of time in an interval just after the ablation to calculate the scale parameter (Figures S7C and S7D). Details of this calculation are in the “scale parameter selection in vivo” of the main text.

### Method details

#### Applying ForSys to *in vivo* zebrafish microscopy

A basal z-slice was selected for each frame of a time-lapse video microscopy. Each frame was segmented with EPySeg^13^ and then manually curated by loading the complete z-volume into tissue analyzer.^47^ The resulting images are skeletonized segmentations of cell membranes, where pixels can only take the value of 1, for membranes, and 0 otherwise. The skeletons are 8-connected and each white pixel has exactly two neighbouring white pixels except for tricellular junction pixels, which have three. The skeleton images can be directly used as input to ForSys Skeleton() function. A preliminary run of ForSys on the segmented images was used to diagnose the accuracy of the tracking for tricell junctions from one frame to the next. Then a JSON file was created to correct any mistracking by labelling the connections between timepoints. As described in the STAR Methods section “determination of the scale parameter in silico”, we used the estimated value of ρ=0.1 for the scale parameter in the dynamic modality. Finally, ForSys outputs for each time-lapse experiment the stress and identity of the membranes, as well as the pressure and identity of the cells in the system.

#### The conceptual model behind ForSys

ForSys uses microscopy images as input to estimate the mechanical state of the tissue. The software extracts vertices, edges, and cells from the segmentation. ForSys accepts skeletonized segmentation, such as those generated with EPySeg^13^ as well as labeled masks, generated for example with Cellpose.^14^ After ForSys reads the input data, the algorithm works identically regardless of the microscopies’ input format (Figure S4). Although most vertices separate two edges, a number of them connect three or more edges and are central for stress inference. We call these pivot vertices or junctions. ForSys calculates the mechanical stresses operating on each edge while assuming mechanical equilibrium in each vertex. Conveniently, this creates a system of equations representing the geometrical state of the tissue.^10^^,^^12^ One equation per coordinate is built from every pivot vertex using force balance at the junction.

In the dynamic modality, ForSys assumes that each vertex is in an overdamped regime, where a viscous damping force proportional to the velocity balances the mechanical stresses at that vertex. This creates a non-homogeneous system of equations where the inhomogeneity is proportional to the vertex velocity. In both static and dynamic modalities, the resulting system of equations is solved through a Least Squares minimization with the constraint that the average tension equals one (see “solving the system of equations” in STAR Methods for more details).

Finally, ForSys uses the stress inferred as an input to estimate cellular pressure within the tissue. For this, a Young-Laplace equation is built at each cell-cell membrane, and the corresponding system is solved similarly to the stresses. However, this requires that the mean pressure of the system is equal to zero (see section statical stress inference for details). ForSys renders intercellular stresses as a color code of the cellular outlines, specifically at the edges. Similarly, intracellular pressures are depicted in a color code within the cytoplasmic area of the cells. Moreover, the numerical values of the inference and other observables are easily exportable, facilitating further analysis of the mechanical state of the tissue.

#### seAPIpy: Generation of *in silico* tissues to validate ForSys

To validate the accuracy of ForSys, we compared the intercellular mechanical stresses inferred by the software with a ground truth distribution of stresses within the tissue. To establish the ground truth, we employed a cell-based computational model to simulate tissues with known intracellular pressures and intercellular stress patterns. Specifically, we employed the vertex model, which is particularly suitable for mechanically evolving epithelial tissues.^53^^,^^54^ For the implementation of the vertex model, we utilized Surface Evolver software.^32^ To facilitate the integration and streamline the simulation process, we developed a Python-based software called seapipy.^33^ This open-source computational tool enables Python scripting to generate the desired initial tissue conditions and simulate them using a vertex model implemented in Surface Evolver. seAPIpy generates a Voronoi tessellation with a given geometry as a starting configuration and assigns initial stresses to the edges (Figure S3). Through seAPIpy functions, the user may add Surface Evolver commands to create the desired conditions for evolution and generate the Surface Evolver-compatible file.

By leveraging both ForSys and the capabilities of seAPIpy software, we implemented four conditions as examples that were later used to test ForSys stress inference in both its static and dynamic modalities. The first two conditions induce a furrow formation on vertical and horizontal strips, respectively. In the third condition, a central zone of elevated stress is introduced, which diminishes radially. Lastly, a fourth condition assigns five different random stresses to edges, following a uniform distribution, with a 50 % spread in stress values. Each condition underwent twenty-five repetitions. These simulations served as the ground truths for validating ForSys *in silico*, as shown in the following two sections (Figures 2A, 3A, and S3).

We generated four examples to validate our software *in silico*. In all four cases, tissues evolve until a time zero is defined. The stresses are modified according to a prescribed condition, and the tissue evolves for shorter periods while it relaxes.

We generated the initial condition in each example by creating a Voronoi tessellation from N = 64 points in a rectangular grid. Each point in the grid is moved with a Gaussian noise centered at zero. Initial cell target areas are randomly assigned as A = 450 ± 5 (mean ± std) from a normal distribution. The initial stress of each edge is also taken from a normal distribution centered at 1 with a standard deviation of 0.1. From this state, the tissue evolved through several rounds of vertex averaging and T1 swaps with varying scales.

We defined time as the number of steps elapsed, multiplied by the scale (t=nΔt), and call it Surface Evolver Time (SET). The first time point is generated after 3875 SET, after which the tissue is evolved for an additional 125 SET. At this point, membrane stresses are changed according to each condition, and each simulation snapshot is saved every 0.25 SET.

In the conditions corresponding to the horizontal and vertical furrows, the new tensions are generated by summing the value corresponding to the position of the center of an edge in the probability density function (PDF) of a normal distribution to the initial randomized value. The normal distribution has its maximum at the centroid of the tissue and a standard deviation of ∼2 cellular radii. Vertical furrows have the PDF on the y-axis, and horizontal furrows on the x-axis. Similarly, the circular furrow uses the distance of the edge’s center to the tissue’s centroid to calculate the new stress. Finally, in the condition corresponding to the “random examples”, tensions are assigned from normal distributions with a 50% spread around five possible values (1, 1.1, 1.2, 1.3, 1.5) chosen uniformly. Cellular pressures are calculated by Surface Evolvers as Lagrange multipliers, by taking into account the area constraints of each cell.

Therefore, seapipy facilitates testing multiple *in silico* examples and has an easy integration into the analysis pipeline. seapipy offers advantages over an existing package (python-evolver; https://github.com/elmisback/python-evolver) because it incorporates Surface Evolver syntax directly into the Python code, eliminating the need to write Surface Evolver commands manually into the input files. seapipy allows for a systematic and straightforward generation of *in silico* ground truths, enabling a better exploration of strengths and limitations in stress inference tools.

#### Statical stress inference

We assume a 2D tissue with C cells, representing each cell as a polygon. The system consists of *V* vertices and *E* edges in total. Each edge is composed of two vertices. We define pivot vertices as those that correspond to junctions between three cells or are at the border of the tissue. This method can be applied to junctions shared by more than three cells but at the risk of losing stability in the underlying model.^55^ All vertices between two pivots are regarded as virtual, and only pivot vertices are used to compute stresses. We then use Newton’s second law and assume mechanical equilibrium to assert that the sum of forces at each pivot vertex equals zero. We can calculate the force acting on each vertex as a sum of the contributions of the forces along the edges connected to it. Mathematically, the force at each pivot vertex will have an equation in the form

(Equation 2) $$\vec{F_{i}}=\sum_{<ij>}\lambda_{ij}\;{\vec{r}}_{ij}$$

Where *i* and *j* indicate the vertices *i* and *j,* $\vec{F_{i}}$ is the force on vertex *i*, $\lambda_{ij}$ is the edge force modulus in that edge, and ${\vec{r}}_{ij}$ is the versor along the edge starting at vertex *i*. The sum is done over all *j* vertices connected to the vertex *i*. Note that $\lambda_{ij}{=\lambda }_{ji}$. The direction of the $r_{ij}$ versor is obtained by fitting a circle to the corresponding membrane, following other authors.^10^^,^^12^

Applying Equation 2 to all the vertices in the tissue will translate into a homogeneous set of linear equations that have to be solved simultaneously with the edge tensions ($\lambda_{ij}$) as unknowns. Hence, we write Equation 2 and equate it to zero for each system vertex to guarantee that all the forces are balanced. Each of these *V* equations will be written as

(Equation 3) $$\lambda_{i1}\;{\vec{r}}_{i1}+\lambda_{i2}\;{\vec{r}}_{i2}+...+\lambda_{E}\;{\vec{r}}_{iV}=0$$

this equation corresponds to the *i*th vertex, and the edge tensions λ are the unknowns.

Similarly, it is possible to infer the pressures of each cell in the tissue by assembling a system of equations that connects the stress at each membrane with its curvature. The Young-Laplace equation relates these quantities with the pressure difference between two neighboring cells. Symbolically,

(Equation 4) $$P_{j}-P_{i}=\lambda_{ij}\rho_{ij}$$

Where *P*_i_ is the pressure of cell *i*, $\lambda_{ij}$ is the stress of the membrane shared by cells *i* and *j*, and $\rho_{ij}$ is the curvature of the shared membrane. This leads to a system of *E* equations, one per edge, and *C* unknowns.

#### The dynamic inference case

The static inference algorithm assumes that vertices do not move. To perform stress inference in a dynamic tissue where vertices are moving, we modified the static algorithm to include vertex movement. If the system has a low Reynolds number, viscous forces dominate the dynamics over inertial components; Equation 2 can be modified, assuming a constant viscosity throughout the tissue, to incorporate viscous forces as

(Equation 5) $${\vec{F}}_{i}=\sum_{<ij>}\lambda_{ij}\;{\vec{r}}_{ij}-\eta {\vec{v}}_{i}$$

where ${\vec{v}}_{i}$ is the velocity of vertex *i* and η is the viscous damping coefficient of the tissue. This would modify the coupled system of equations, which could be rearranged to get the ith vertex

(Equation 6) $$\sum_{<ij>}\lambda_{ij}\;{\vec{r}}_{ij}=\eta {\vec{v}}_{i}$$

To determine the scales correctly, we proceeded to make Equation 6 nondimensional. For this, we redefine the stresses by using an unknown reference stress $\lambda_{j}^{\prime }=\frac{\lambda_{j}}{{\bar{\lambda }}_{j}}$. We take this reference stress as the average stress in the system. We used a reference velocity defined as the time average over all the frames of the mean junction velocity

(Equation 7) $$\bar{v}=\frac{1}{N_{frames}}\;\sum_{t=t_{i}}^{t_{f}}\frac{\sum_{i}\left\|{{\vec{v}}_{i}}^{t}\right\|}{N_{v}^{t}}$$

Combining these equations gives a nondimensional expression for the force balance at each junction

(Equation 8) $$\sum_{j}\lambda_{j}^{\prime }{\vec{r}}_{ij}=\left(\frac{\eta \;\bar{v}}{\bar{\lambda }}\right)\;\frac{{\vec{v}}_{i}}{\bar{v}}$$

Importantly, this led to the nondimensional parameter $\frac{\eta \;\bar{v}}{\bar{\lambda }}$. Even though the right-hand side of Equation 5 is not a viscosity but rather a damping coefficient, we can interpret it as such in this context. Therefore, as the stress of each membrane represents the elastic forces in the system, this parameter can be interpreted as the relation between the elastic and the viscous forces acting on the system. We call this number the scale parameter, *ρ* ($\rho =\frac{\eta \;\bar{v}}{\bar{\lambda }}$).

#### Solving the system of equations

In static and dynamic cases, it is necessary to solve a system of linear equations with homogeneous and inhomogeneous conditions, respectively. In both cases, we will turn the system into its matrix form, add a constraint to the unknowns through a Lagrange multiplier, and convert it into a least squares problem. Finally, we will attempt to invert the resulting matrix, and if that is not possible, we will use a numerical algorithm to find the best solution.

Given a two-dimensional tissue with *V* vertices and *E* edges, the system would have 2*V* equations, as each vertex has one equation per dimension and *E* unknowns, one for each edge. Following the method proposed by Brodland et al.,^10^ the set of equations in Equation 2 is then translated into matrix form as

(Equation 9) $$M_{\lambda }X=B$$

Where $M_{\lambda }$ is a 2*V* x *E* matrix with versor coefficients, *X* is the unknowns column matrix of *E* x 1, and *B* is a 2*V* x 1 column matrix with either all zeros under static conditions or the velocity components for each vertex in the dynamical case. To avoid the null solution in the static case, one further condition is added: The mean value for the unknowns, *i.e.*, *λ*s, is set equal to one, using the equation

(Equation 10) $$\sum_{k=1}^{E}\lambda_{k}=E$$

where *E* is the number of edges and $\lambda_{k}$ is the tension corresponding to the *k*th edge. In the matrix representation, this entails adding a Lagrange multiplier to the unknowns, a row and column of ones for the tension constraint, and a new row in the *B* matrix. Hence, the equations to be solved have 2*V* + 1 equations and *E* + 1 unknowns. Therefore, the typical equation for the one of the components of vertex *i* will be of the form

(Equation 11) $$\lambda_{ij}r_{ij}^{x}+\lambda_{ik}r_{ik}^{x}+\lambda_{il}r_{il}^{x}+L=\left(\frac{\eta \bar{v}}{\bar{\lambda }}\right)\frac{v_{\;i}}{\bar{v}}$$

where *j*, *k* and *l* are vertices that connect to vertex *i*, and *L* is the Lagrange multiplier added by the constraint on the average value of stresses.

As this system might not always guarantee a solution, we transformed it using least squares. To this end, we apply the transpose matrix $M_{\lambda }^{tr}$ to the equation, giving a new system

(Equation 12) $$M_{\lambda }^{tr}\;M_{\lambda }X=M_{\lambda }^{tr}B$$

Symbolically,

(Equation 13) $$M_{\lambda }^{\prime }\;X^{\prime }=B^{\prime }$$

On the other hand, the *B* matrix in Equations 6 and 7 for the dynamic case has the corresponding nondimensional velocity component multiplied by the scale parameter (ρ) described in Equation 8 in each row. Its final element has the number of edges *E* to enforce the constraint. To quantify the movement present in the tissue, we calculate the 2-norm of the *B* matrix, removing the last row, this vector is referred to as $|v{|}_{2}$. Each vertex is tracked through time to obtain the vertex velocity, and the forward velocity is calculated in all but the last step, where the backward expression is used. If a vertex cannot be followed in a frame, i.e., due to significant changes in the tissue shape, it is assigned a null velocity for the frames where it cannot be tracked.

Hence, to elucidate the acting forces within the tissue, the software attempts to solve it by inverting the ${M_{\lambda }}^{\prime }$ matrix, thus having a solution

(Equation 14) $$X={M^{\prime }}^{-1}B^{\prime }$$

If the system is not invertible, *i.e*., *M*’ is singular, or if any of the edge tensions found are negative, a Least Squares algorithm can be used to find the stress values, such as a Non-Negative Least Squares, SciPy’s package or lmfit.^56^^,^^57^^,^^58^

After solving the system, the calculated stresses can be used to infer the pressures of the cells. As seen from the Young-Laplace equation (Equation 4), pressures are expressed through an inhomogeneous system of linear equations. The left-hand side is a matrix with one column per cell and one row per membrane. Each row has two entries different from zero, one +1 and one -1, representing the difference in pressure at that membrane. The right-hand side consists of a column matrix with the product of each membrane’s stresses and curvatures ($\lambda_{ij}\rho_{ij}$). Then, the equations are solved analogously to the stress case using the Least Squares with the constraint that the average pressure must be zero.

#### Determination of the scale parameter *in silico*

We performed a parameter sweep to determine the optimal value of the Scale Parameter (*ρ*) from *in silico* simulations, testing values ranging from 0 to 0.5. For each of the four simulation scenarios, and across five repetitions per scenario, we calculated a performance score at each time point (Figures S9A–S9D). The optimal value of *ρ* was selected as the median score across repetitions, which in each case also coincided with the mode (Figures S9E and S9F). The resulting values were: *ρ* = 0.08 for the x-axis furrow, *ρ* = 0.07 for the y-axis furrow, *ρ* = 0.13 for the circular furrow, and *ρ* = 0.18 for the random tension configuration. Based on these results, we set the default scale parameter to 0.1 in the dynamic inference mode of our software.

#### Interacting with the ForSys software

Forsys requires a Python (>3.8) environment to run. After installation, there are three ways to use the ForSys software. First, writing a Python script is the most comprehensive method, giving full access to all the information that ForSys processes. Example scripts can be found in the documentation, and the packages repository, and in Figure S1. Additionally, to improve the usability of the ForSys tool, we created a Command Line Interface (CLI), and a Graphical User Interface (GUI). The CLI allows the analysis of tissues, using dynamical or statical inference directly from the command line, without needing to write any Python Scripts (Figures S2A and S2C). Possible commands to use are detailed in the package’s documentation. Finally, a GUI was created by integrating ForSys to Fiji to allow a high level interaction with the software (Figures S2B and S2C).

### Quantification and statistical analysis

#### Evaluating goodness of fit

We evaluated the goodness of fit of the inferred data to the ground truth using a tailored saturated score function. This score combines the Pearson correlation coefficient (p), the Mean Absolute Percentage Error (M), and the coefficient of determination (r) as

(Equation 15) $$s\left(M,p,r\right)=\frac{\alpha }{M}+\frac{\beta }{2}\frac{1+p}{1-p}+\frac{\gamma }{1-r}$$

where ⍺, *β* and ɣ are free parameters set to one. As this function is unbounded from above, we saturate the score at s = 299.5 for representation purposes in Figures 2, 3, and S9. This value comes from an error of 1 %, *i.e.* s(0.01,0.99,0.99)=299.5.

The three components of the score are displayed separately for the *in silico* static validation of ForSys in Figures 2D, S6A, and S6B, and for the dynamic validation in Figures S7A–S7C.

#### Statistical estimators

To compare distributions, the Mann-Whitney *U* test was used with different alternative hypotheses, depending on whether we tested for stochastic ordering or whether distributions are different. In all *in silico* cases, the number of samples is twenty-five, which is the number of repetitions per condition. The Pearson correlation coefficient (*R*) was used when we evaluated correlations. The number of samples in each case is indicated when reporting the *p*-value.

#### Comparing ForSys with other computational methods

We tested the similarity of the static implementation of ForSys with two other established software: CellFIT^10^ and DLITE.^12^ To this end, we applied the DLITE python package to solve the four *in silico* examples used throughout this work, taking advantage of its CellFIT modality. We found that inferred stress for the last frame of each of the examples are similar (Figure S5A) and that the stress distributions emerging from the solution are roughly identical (Figure S5B). Moreover, for the accumulated data of all repetitions for each example at the last simulated frame, we found that ForSys static performs better than DLITE, except in the random example (Figure S5C).

Moreover, we generated an artificial normal distribution to measure the relative differences with a first moment of 1 and a second moment equal to 0.2. We calculated the Wasserstein Distance between the *in silico* distributions and the normal generated randomly. Given two distributions, X and Y, the Wasserstein Distance is zero if and only if the two distributions are equal. The distance between two distributions can be arbitrarily large for increasingly different shapes.

The Wasserstein Distance is almost zero in all cases, indicating that the distributions gathered from the three inference methods are similar. To compare its similarity, we used an artificially generated normal distribution. Using this metric, we found that the methods among themselves are ∼30 times closer in the x-furrow and y-furrow, 10 times closer for the circular case, and ∼5 times closer in the random densities example than to the normal distribution.
