# Supplementary material for: ForSys: Non-invasive stress inference from time-lapse microscopy
Source: iScience. 2025 Oct 3;28(11):113685. doi: 10.1016/j.isci.2025.113685 (PMC12556342; doi:10.1016/j.isci.2025.113685)
Supplement: Document S1. Figures S1–S9 [file mmc1.pdf]

**iScience, Volume *28***

## Supplemental information

# ForSys: Non-invasive stress inference

# from time-lapse microscopy

**Augusto Borges, Jerónimo R. Miranda-Rodríguez, Alberto S. Ceccarelli, Guilherme Ventura, Jakub Sedzinski, Hernán López-Schier, and Osvaldo Chara**

# Supplementary Figures

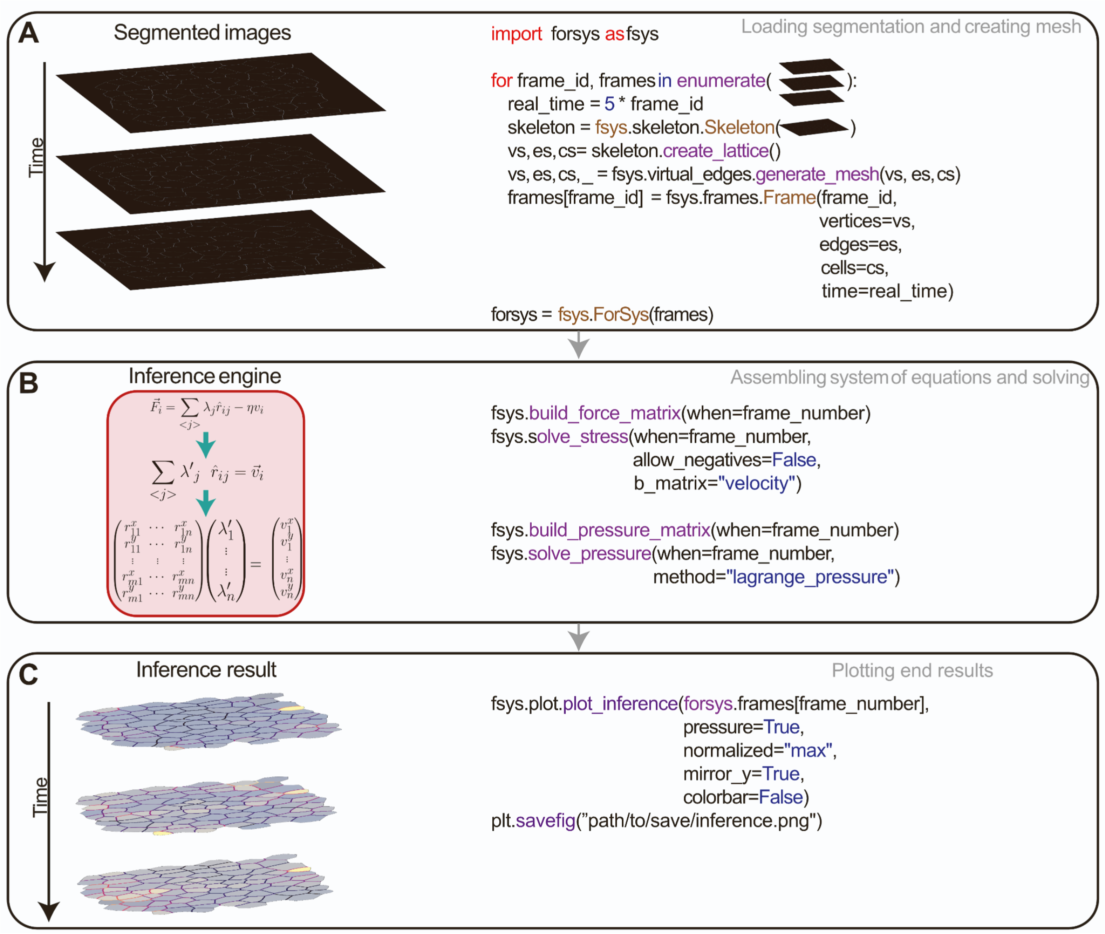


**Figure S1. ForSys general implementation, relates to Figure 1.** Forsys uses segmented images as input, which are read with the Skeleton() module of the software. This module allows binary tiff masks, as well as labeled masks as input. Frames are collected in a dictionary and then passed to the ForSys() class to generate the corresponding mesh **(A)**. Then, the matrices for the stresses and the pressures are built through different modules and solved individually **(B)**. Stresses must be previously calculated to infer the pressures due to their dependence on the membrane stress. Finally, the inferred stresses and pressures are exported through the *plot_inference()* methods **(C)**.

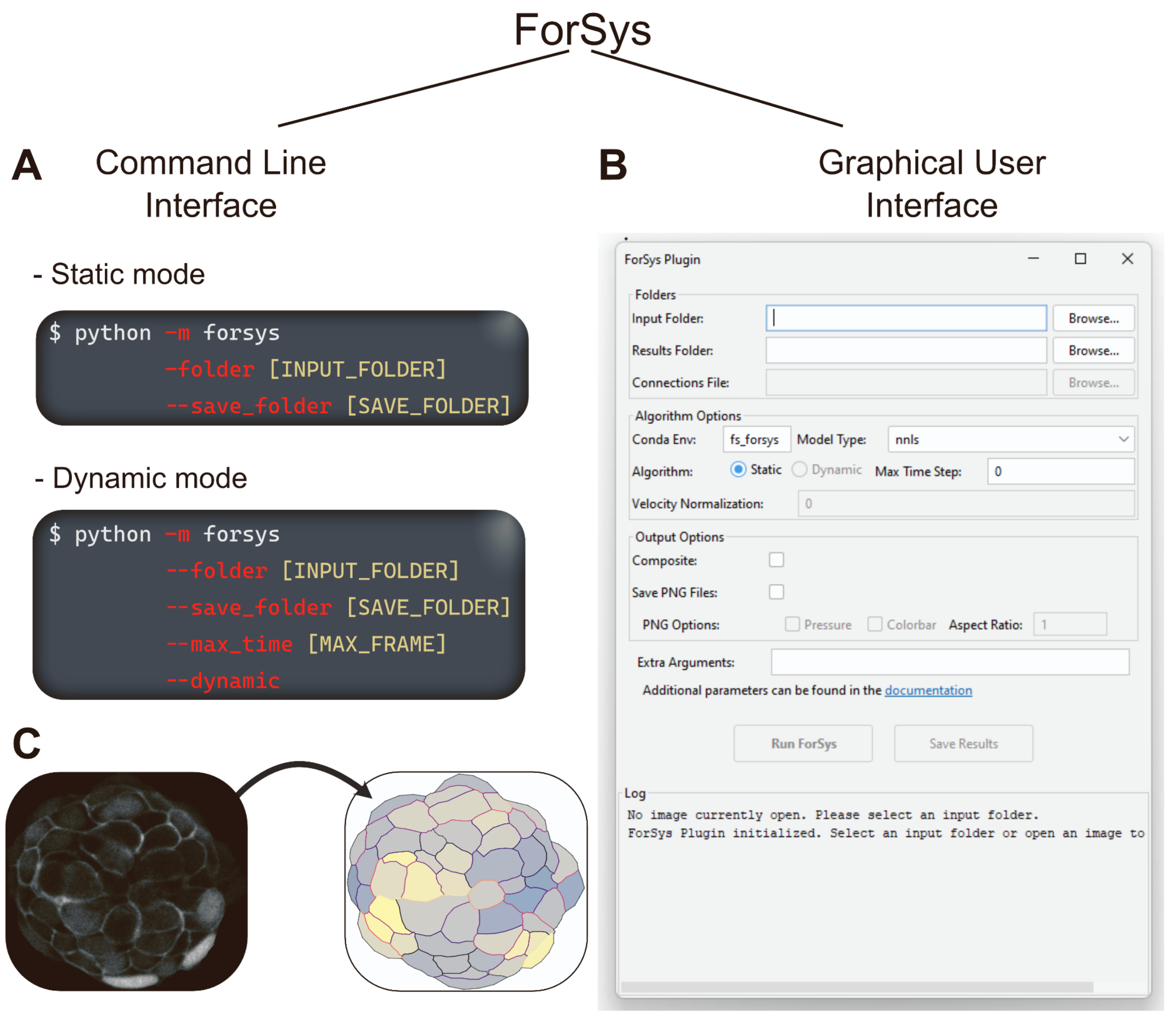


**Figure S2. Using the ForSys software, relates to Figure 1.** Aside from Python scripting, ForSys can be used through a Command Line Interface or a Graphical User Interface, implemented in Fiji. **(A)** The command line interface allows both Static and Dynamic modality to run through simple command flags. Moreover, Fiji allows ForSys to be run through a graphical interface **(B)**. Below a zebrafish neuromast is shown as an example **(C)**. All additional information can be found in the ForSys documentation.

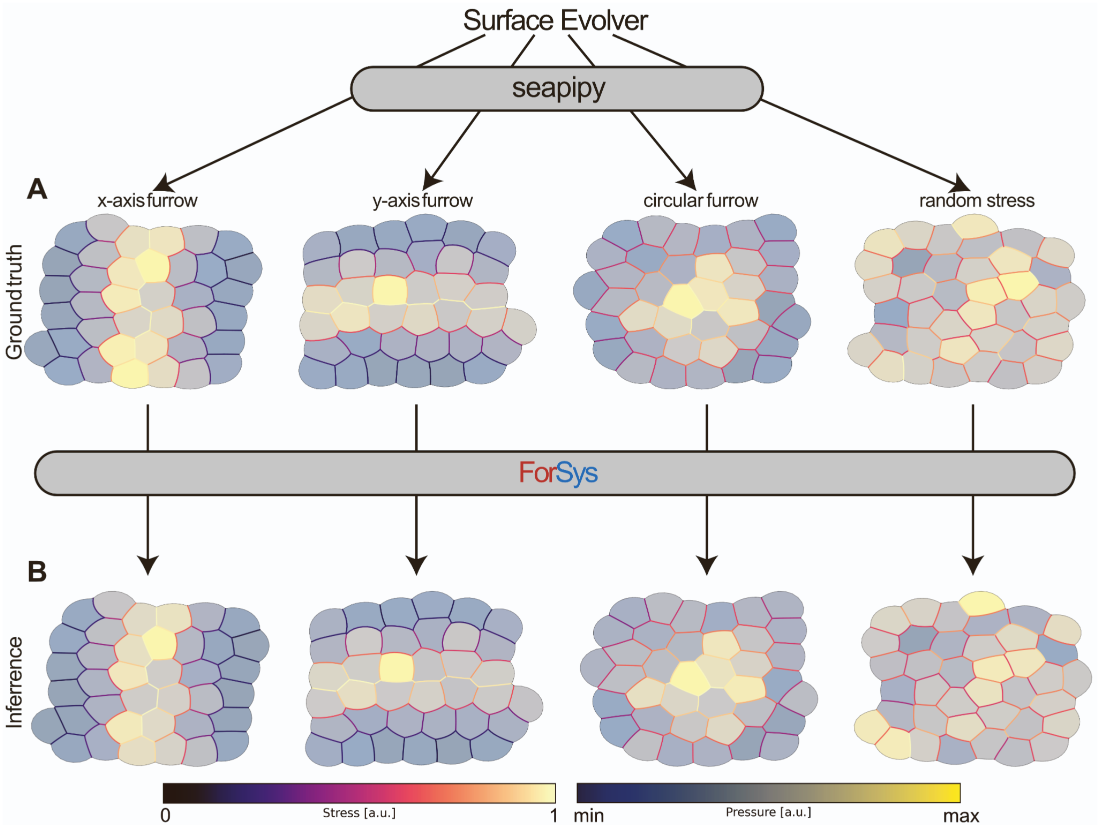


**Figure S3. ForSys pipeline for validation, relates to Figure 1.** We used different conditions to generate example tissues with varied stresses and pressures. All examples are created through the seapipy package, which uses Surface Evolver as a backend **(A)**. Then, ForSys is applied in any of its modalities to infer the stresses and pressures **(B)**.

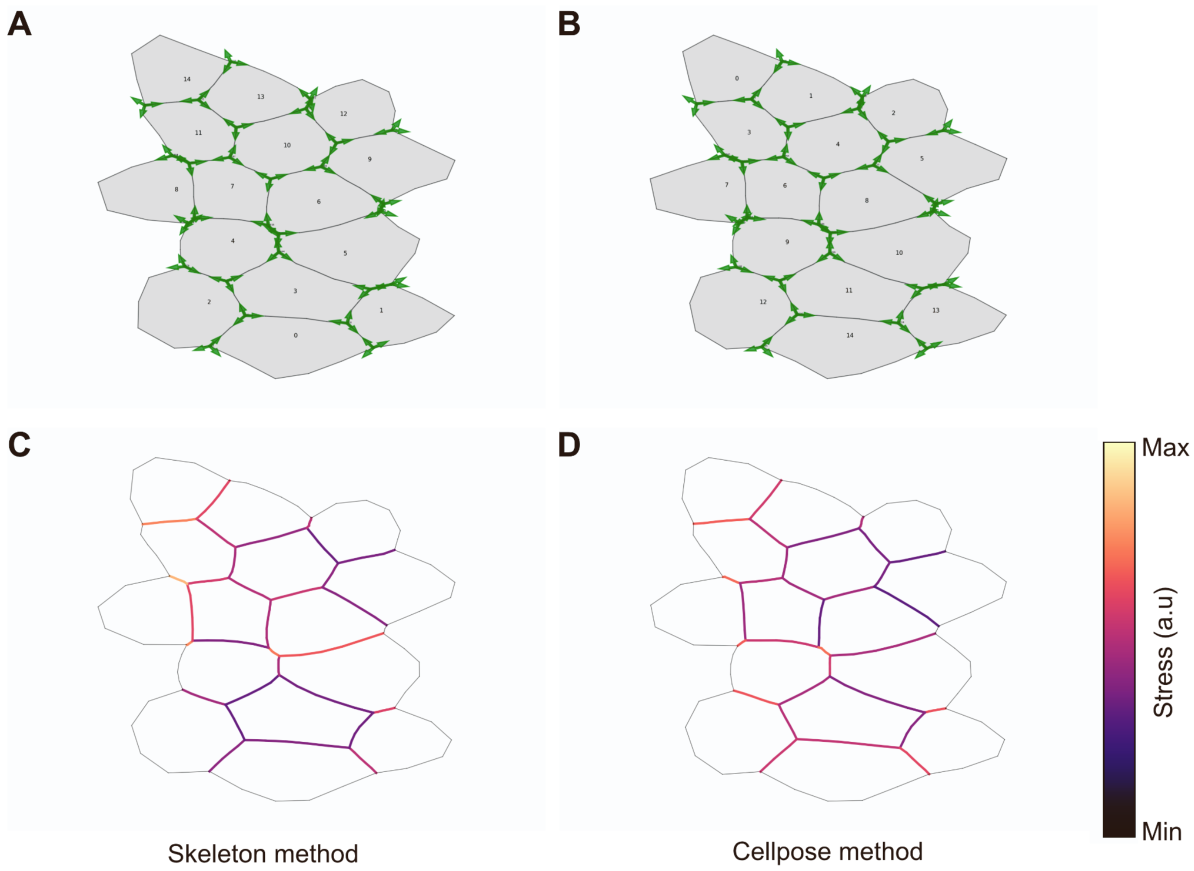


**Figure S4. Input formats, relates to Figure 1.** ForSys accepts skeletonized binary masks such as the ones generated by EPySeg [13], as well as labeled masks, such as those generated by Cellpose[14]. In both cases the algorithm, after the initial reading of the input files, proceeds identically. **(A)** and **(B)** shows the vector arrows resulting from the circular fit to each of the membranes in all tricellular junctions for the input based on the Skeletonized format, and the Cellpose format, respectively. There are no observable quantitative differences between the tissues. Paneles **(C)** and **(D)** show the predicted stress for the given tissue for Skeletonized and Cellpose, respectively. Some changes are expected in the stress patterns due to the different segmentations, however results differ only slightly between both input methods. In the figure, the microscopy used corresponds to the Embryo 2 in Figure 4B.

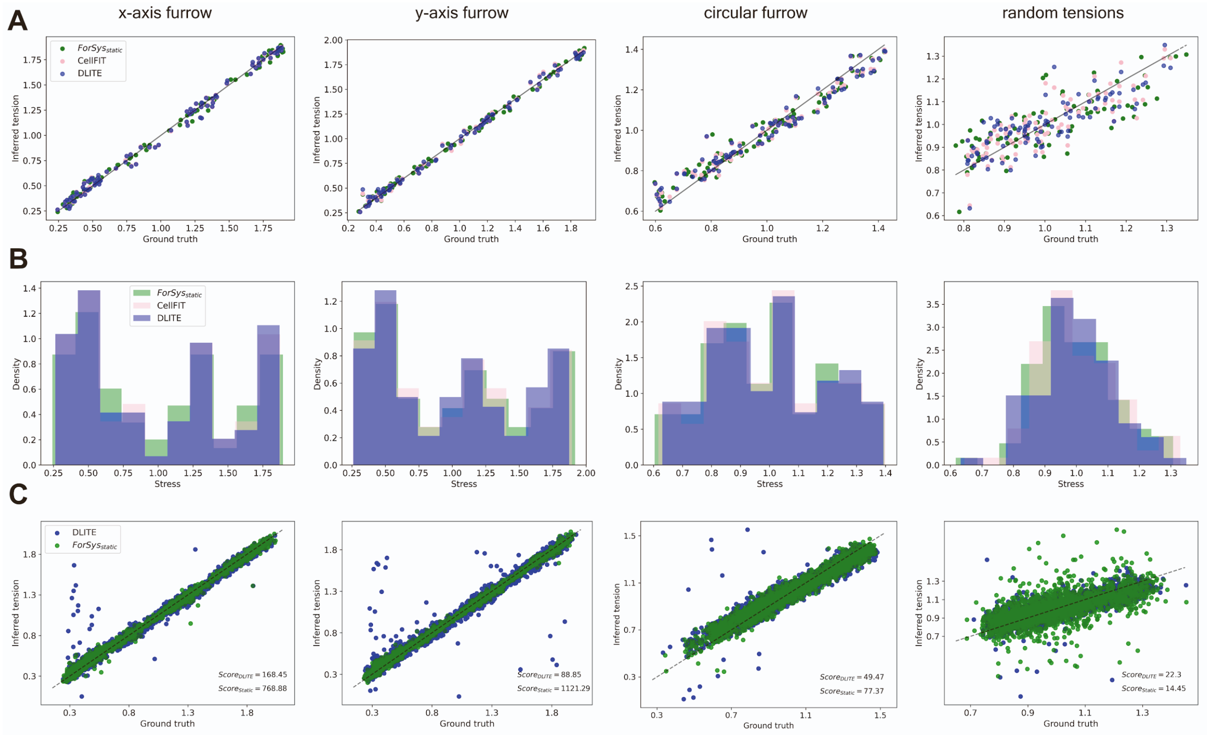


**Figure S5. Comparison between static ForSys and other force inference methods, relates to Figure 2.** We tested whether the static implementation of ForSys (green) differed from the values of DLITE (blue) and CellFIT (pink). Each column represents one of the examples. We show that the inferred stress versus the ground truth follows the y=x line, plotted as a solid black line as a visual aid, for the three methods at the last simulated frame **(A)**. Moreover, the distribution of stresses of all methods has similar behaviors in the histograms **(B)**. Both panels **(A)** and **(B)** are for a selected representative simulation. Then, the result for all inferred tensions versus ground truth repetitions is shown for each condition at the last simulated frame. The black dashed line is the y=x line and is a visual aid. The score function's values are in the lower right corner of each plot **(C)**. ForSys, in its static modality, has better results in the three first examples and comparable results in the random tension case.

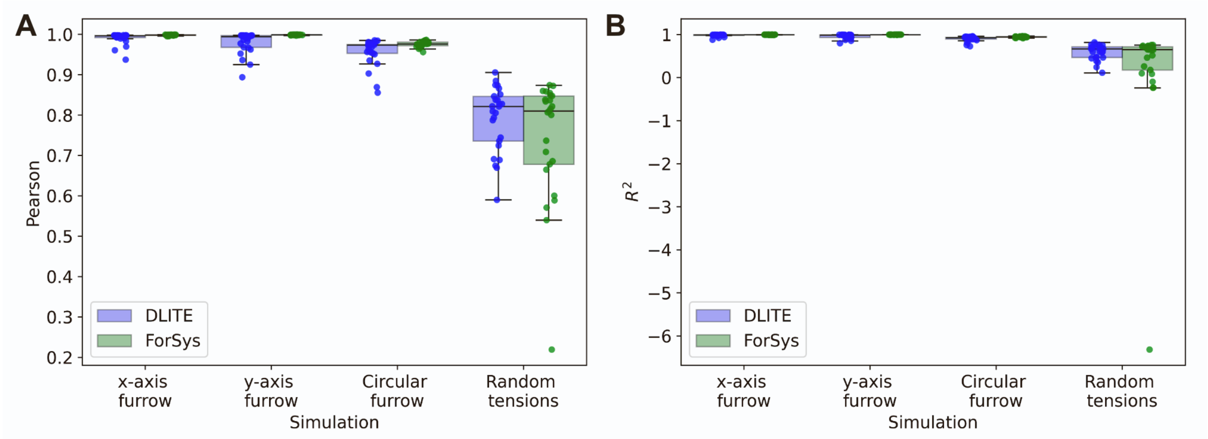


**Figure S6. ForSys static score components, relates to Figure 2.** The Pearson correlation coefficient (A), and $R^2$ value (B) are shown. These values, along with the Mean Absolute Percentage Error (MAPE) in Figure 2D, are used to calculate the Score function in Figure 2E.

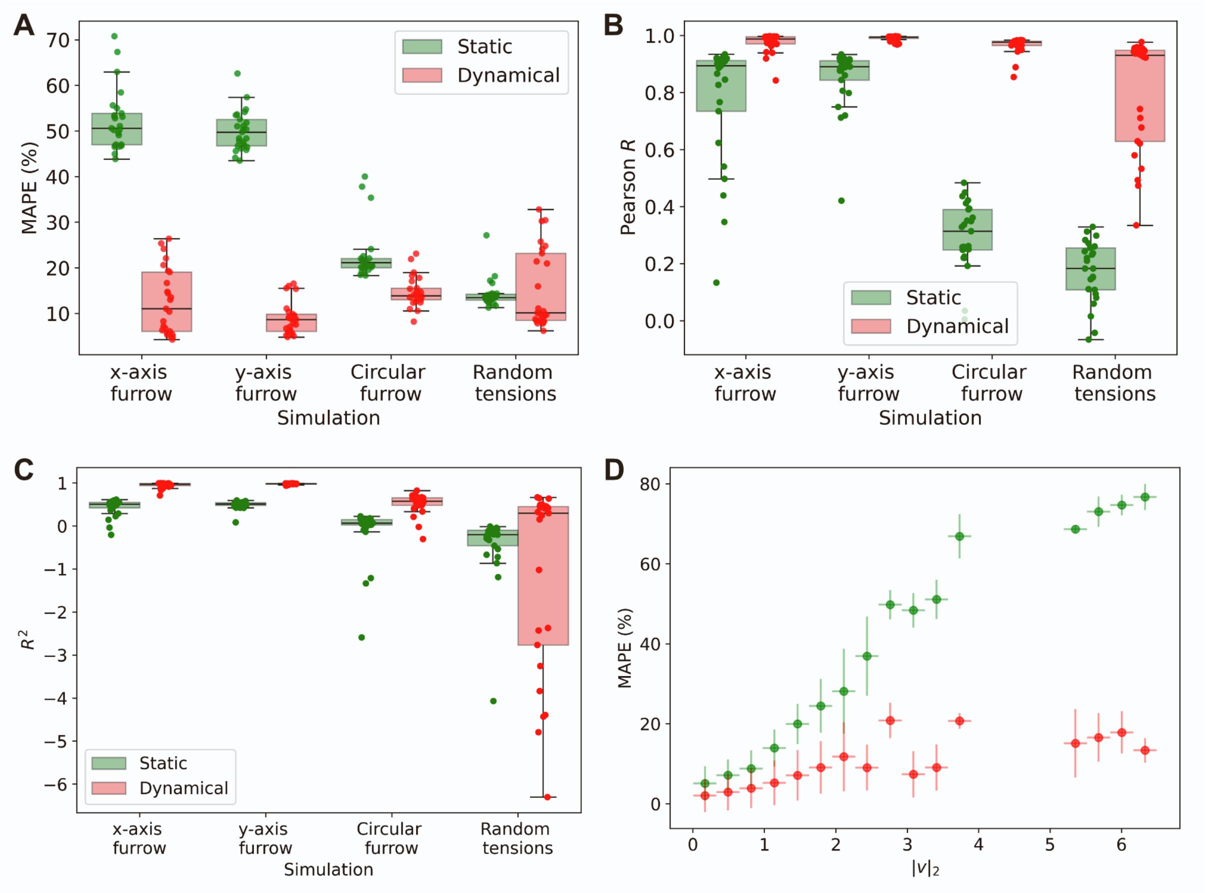


**Figure S7. ForSys dynamic Score components and velocity dependance, relates to Figure 3.** The Mean Absolute percentage Error **(A)**, Pearson correlation coefficient **(B)**, and $R^2$ value **(C)** are shown. These values are used to calculate the Score function for the inferences performed to validate the dynamic modality of ForSys *in vivo*, that is shown in Figure 3D. **(D)** Dependence of the MAPE with the velocity $|v|_2$. The scattered dots are the median for all experiments with a velocity corresponding to the current $|v|_2$ bin. Error bars in the y-axis are one standard deviation, and error bars in the x-axis represent the size of the velocity bin. The green color represents static modality, and red color dynamic modality.

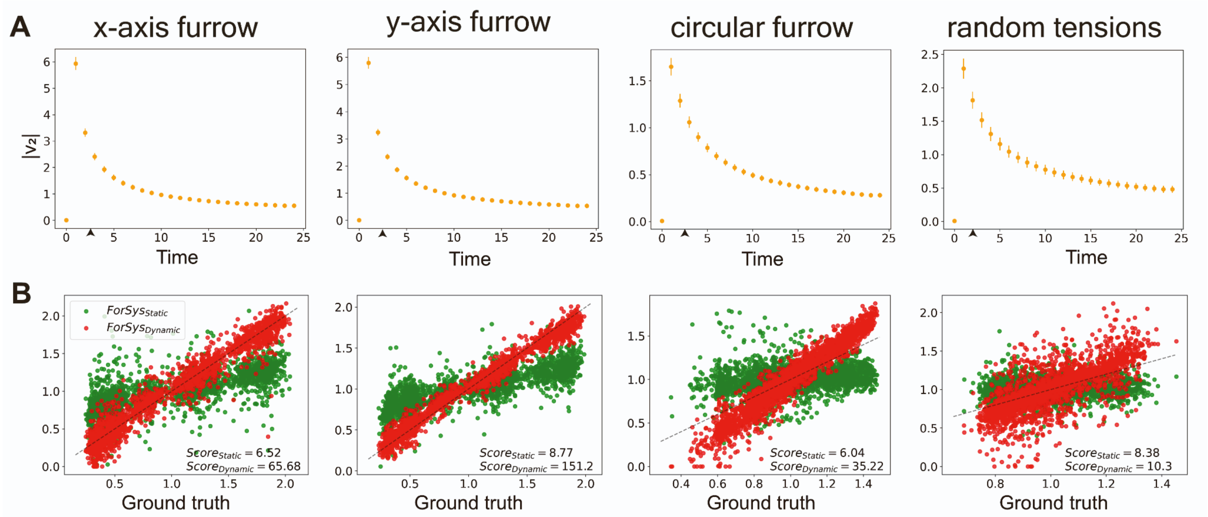


**Figure S8. Forsys dynamic at each example, relates to Figure 3.** Each column groups plots corresponding to the same prescribed conditions. **(A)** The evolution of tissue movement for each example is shown. The orange scatter dots are the mean of the 2-norm of the velocities vector, with the uncertainty being one standard deviation. In all cases, the values are derived from each example's repetitions. **(B)** The inferred tension versus ground truth is plotted for all examples. Dynamical results are plotted in red, while static ones are in green. The y = x is plotted as a visual aid as a dashed black line. The score function values are in the lower right corner of each plot. In every case ForSys in its Dynamic modality gives a better score than its static counterpart.

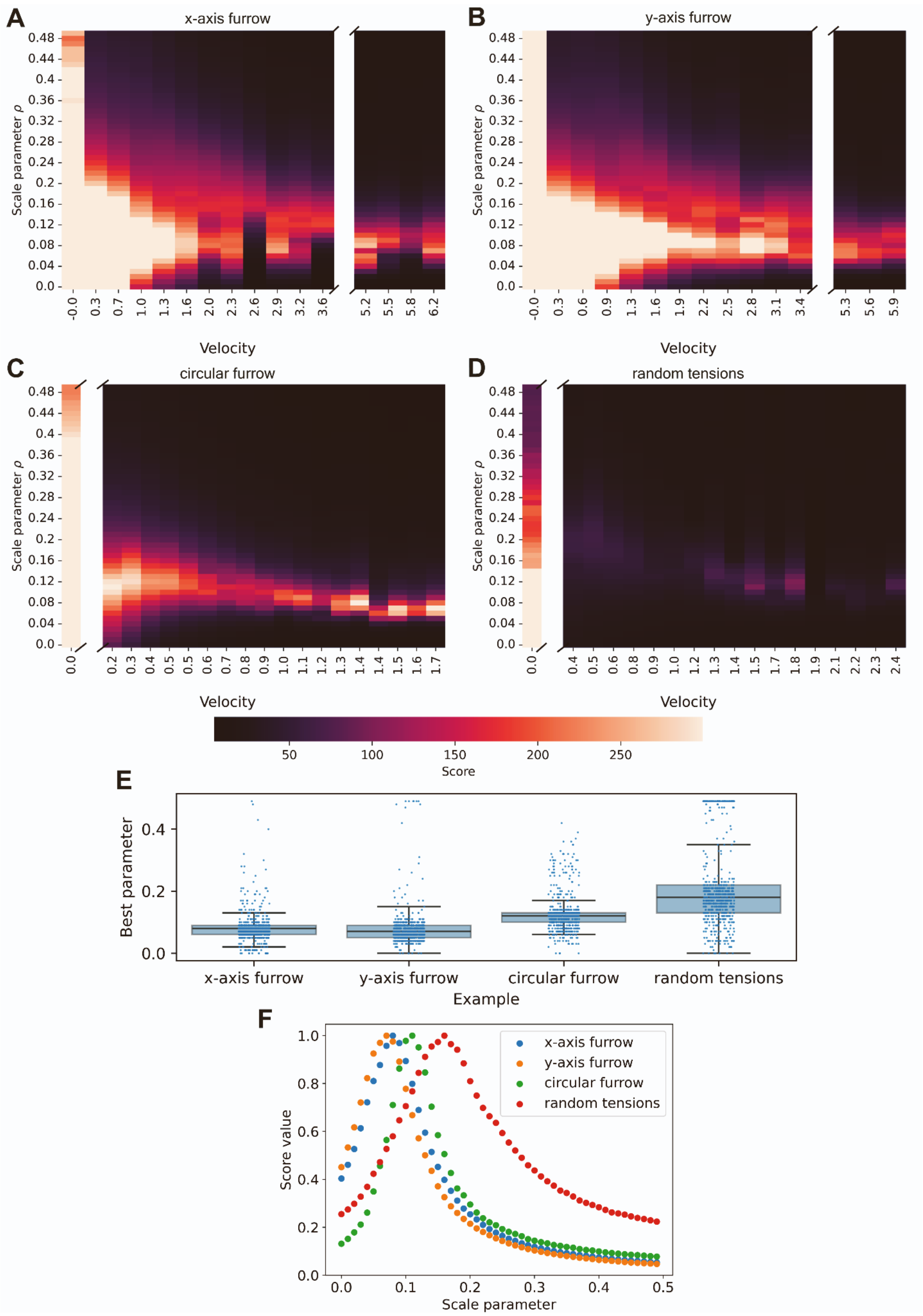


**Figure S9. Scale parameter exploration, relates to Figure 3. (A-D)** Heatmaps show the saturated score function values for the sweep of the scale parameter ρ for each example and all 25 repetitions as a function of the

velocity of the frame. This velocity is defined as the norm of the vector containing the velocities in the x and y direction of each tracked junction in the tissue. The score function is calculated as described in Materials and Methods, with saturation at the corresponding value $s(0.01,\ 0.99,\ 0.99)$. **(E)** Shows the boxplots for the scale parameter corresponding to the highest score value for each time point and example. The median values of these four distributions are $m_{x-axis} = 0.08; m_{y-axis} = 0.07; m_{circular} = 0.12; m_{random} = 0.18$. The corresponding examples for Figures 2 and 3 use these values as scale parameters. **(F)** The median value of the score for the 25 simulations per prescribed condition is shown as a function of the scale parameter value. Higher Fit score signifies a better agreement between inference and ground truth. Scores are normalized to the maxima of each curve, to make them comparable.
